# Rapid detection of Kenyan tomato leaf curl virus isolates using probe-enhanced loop-mediated isothermal amplification coupled with a modified DNA extraction method

**DOI:** 10.1371/journal.pone.0349665

**Published:** 2026-05-22

**Authors:** Abigarl Ndudzo, Florence Ng’ong’a, Edith K. Avedi, Elijah M. Ateka

**Affiliations:** 1 Department of Molecular Biology and Biotechnology, Pan African University Institute of Basic Sciences, Technology and Innovation, JKUAT, Kenya; 2 Department of Biotechnology, Faculty of Environmental and Life Sciences, Lupane State University, Zimbabwe; 3 Department of Biochemistry, Jomo Kenyatta University of Agriculture and Technology, Kenya; 4 Phytosanitary and Biosecurity Department, Kenya Plant Health Inspectorate Services, Kenya; 5 Office of the Dean, School of Agriculture and Environmental Sciences, Jomo Kenyatta University of Agriculture and Technology, Kenya; Pan African University of Life and Earth Sciences Institute, PAULESI, NIGERIA

## Abstract

Plant disease management requires sensitive and user-friendly diagnostic methods. The polymerase chain reaction (PCR) and other methods have helped diagnose diseases, but they are primarily used in high-resource settings, leaving low-resource settings without effective options. A simple probe-enhanced method for the detection of Kenyan tomato leaf curl virus (Kenyan ToLCV) isolates in host crops, including asymptomatic ones, was developed by employing loop-mediated isothermal amplification (LAMP) coupled with a rapid modified DNA extraction method. A modified alkaline polyethylene glycol (APEG) approach for sample preparation from infected tomato and chili pepper leaves was optimized for sensitivity, reproducibility, and adaptability. The modified extraction buffer contained polyethylene glycol, sodium hydroxide, polyvinylpyrrolidone, and sodium chloride. Six LAMP primers and a hybridization probe targeting the AV1 gene were designed and used for specific detection of Kenyan ToLCV isolates. Gel electrophoresis, real-time detection, and color change were used to evaluate the LAMP reaction. The probe-enhanced LAMP assay detected the virus in 2.7 minutes under optimal conditions. The analytical sensitivity reached 0.001 fg/μL of total DNA. The optimized LAMP assay was 10^6^ times more sensitive than conventional gold-standard PCR and did not cross-react with other tomato-infecting viruses. The modified buffer yielded a significantly shorter time-to-positive (~4.8 minutes; p < 0.0001) compared to unmodified APEG. The probe-enhanced LAMP assay detected the virus in 50% (4/8) of asymptomatic samples. The findings suggest the application of the developed assay for phytopathological testing in resource-poor areas and for direct field detection. This assay would provide a reliable approach for rapid plant health certification and quarantine inspection.

## 1 Introduction

The current status of plant pathogen detection in Africa indicates a growing recognition of the significance of efficient diagnostic methods to improve agricultural productivity and food security. Viruses are widespread and destructive plant pathogens, responsible for significant economic losses worldwide [1]. The emergence of new virus strains or variants is driven by factors including climate change, evolving farming practices, and mixed infections; this epidemiological complexity underscores the necessity for continuous surveillance [2]. In Kenya, tomato leaf curl virus has been identified as one of the biotic constraints on horticultural production. Recent studies have reported the presence of tomato leaf curl Arusha virus [3] and the tomato leaf curl Uganda virus [4] in tomato plants displaying symptoms of begomovirus infection. The Kenyan ToLCV isolates are monopartite begomoviruses with a circular single-stranded DNA (DNA-A) genome of about 2.7–2.8 Kb, encoding both virion- and complementary-sense open reading frames [3]. For monopartite begomoviruses, the DNA-A component alone is sufficient to cause systemic infection, making tomato leaf curl viruses a major challenge for horticulture. The virus spreads via the whitefly *Bemisia tabaci* and induces leaf curling, yellowing, stunting, and reduced fruit set, causing severe yield losses, especially in Africa’s tropical and subtropical regions [3,4], sometimes reaching 100% losses if infection occurs early [3]. Management of ToLCV is mainly preventive, given its intracellular nature of viral replication [5]. Moreover, ToLCV symptoms may resemble those from various other etiologies, and their manifestation is variable, making symptom-based detection inadequate and unreliable. For smallholder farmers in sub-Saharan Africa, tomatoes are a crucial source of income, and viral diseases reduce yields and food security. Early, precise pathogen detection is critical for monitoring outbreaks and preventing future pandemics [2,6].

Several immunological and molecular methods are used to detect ToLCV. Among serological methods for ToLCV detection are the triple antibody sandwich enzyme-linked immunosorbent assay [7], dot enzyme-linked immunosorbent assay, and direct tissue blot immunoassay [8]. For other viruses, techniques such as western blotting, dot-immunobinding assay, and direct antigen-coating enzyme-linked immunosorbent assay have been used [9]. While these serological methods provide baseline data, they lack the sensitivity needed for early detection or large-scale indexing of plant material [10]. As a result, molecular diagnostics have become indispensable for producing virus-free germplasm and enabling the safe international exchange of breeding lines [11]. Specifically, molecular methodologies, including PCR [12], nested PCR [13], real-time PCR [14], nano-PCR [15], “*amplification-free CRISPR/Cas12a*” [16], and multiplex PCR [17], offer superior sensitivity and specificity. These molecular approaches outperform immunological techniques [18]. Nucleic acid-based methods can detect ToLCV in asymptomatic samples and distinguish closely related species resulting from slight sequence changes [19]. PCR, commonly regarded as the gold-standard, remains the preferred approach for detecting plant diseases due to its exceptional specificity and sensitivity [18]. Despite this efficacy, PCR has practical constraints, including the need for refrigerated enzymes, costly and complex thermocyclers, which limits their use to centralized laboratories [20], and the requirement for skilled technical personnel, which restricts broader adoption [21]. Additionally, co-extracted plant inhibitors compromise DNA quality, non-specific primer annealing reduces specificity, and the inherently long reaction times of PCR further limit its suitability for rapid field diagnostics [22,23]. These combined challenges underscore the ongoing need for more accessible and effective alternatives to PCR-based point-of-care diagnostics.

Isothermal amplification technologies, such as LAMP, are gaining popularity due to their rapid turnaround times and accurate results in point-of-care settings [18,22]. Given Africa’s diverse agricultural environment and the challenges posed by plant viral infections, LAMP shows strong potential for detecting plant pathogens. LAMP uses four to six primers, two outer (F3 and B3), two inner (FIP and BIP), and optionally two loop primers, together with *Bst* polymerase, which possesses strand-displacement activity [24–26], to amplify DNA. This process creates loop regions, allowing additional primers to bind and enabling continuous amplification without thermal cycling. Loop primers hybridize to the loop regions of LAMP amplification intermediates, providing additional initiation sites and thereby accelerating the amplification reaction [27]. The inclusion of a hybridization probe increases specificity and enhances real-time detection [28]. LAMP requires primers to bind up to eight target regions, providing high specificity. It has been used to detect ToLCVs in plants [29–33], and offers sensitivity 10–100 times higher than conventional PCR [20,34], comparable sensitivity to qPCR, and greater tolerance to inhibitors [35]. LAMP is faster (30–60 minutes) than conventional PCR, and it generates large amounts of DNA (approximately 10 μg per 25 μL, compared to 0.2 μg for conventional PCR), enabling innovative detection methods [20,25,27,36]. Simplified product identification and compatibility with crude DNA extracts [34,37,38] further support LAMP’s effectiveness for fieldwork in resource-limited contexts. LAMP is flexible, fast, and suitable for field deployment.

While LAMP is widely used for plant virus detection, sample preparation methods remain highly variable, are rarely optimized, and often lack standardization, especially regarding extraction buffer composition. Isolating and purifying the DNA template is essential for molecular diagnostics, but the effort and time required to prepare DNA samples frequently bottleneck the workflow. Most methods for isolating and purifying DNA templates are arduous, time-consuming, and expensive due to their complexity, the need to handle numerous samples, and their dependence on specific plant types and tissues. An ideal DNA extraction method should reduce the handling of a tissue sample from collection to analysis, increase DNA yield, be versatile across organisms, allow large-scale sample processing while minimizing labor and material requirements, and not produce hazardous waste that could harm the environment [39]. Many plant cell lysis buffers, such as alkaline buffers [40], polyvinylpyrrolidone [41], CTAB [42], and APEG [43], have been used to rapidly isolate template DNA from secondary compounds, such as polyphenols, which bind to DNA even after lysis. Plant pathology requires rapid DNA extraction to detect plant diseases in point-of-care and resource-limited agricultural settings. Most LAMP assays for plant virus detection use crude extraction methods and often rely on simple alkaline buffers, rather than on systematically optimized protocols [33,44]. Field-applicable LAMP assays heavily depend on the effectiveness of the crude extraction method, especially in field or resource-limited settings. However, there has been little effort to systematically optimize extraction buffer composition, which is a major gap in current diagnostic methods. In this study, modifying the alkaline polyethylene glycol method [43] yielded a new plant cell lysis buffer comprised of polyethylene glycol, sodium hydroxide, sodium chloride, and PVP. The modified APEG lysis buffer is expected to enhance efficiency, and reduce labor in resource-constrained agricultural settings. This approach demonstrates versatility, enabling the isolation of DNA from various plant species, including those with elevated polyphenol levels, and the amplification of challenging tissues.

Currently, most African governments face resource limitations in effectively monitoring disease outbreaks in horticultural crops, including tomatoes and chili peppers. Limited resources can hinder surveillance efforts, data acquisition, and timely responses to disease outbreaks, thereby compromising livelihoods and local food security. This gap underscores the need to understand the incidence, magnitude, and consequences of ToLCV outbreaks. The timely diagnosis of pathogen-associated diseases is essential for effective management; however, developing rapid, sensitive, and accurate diagnostic tools remains a challenge [21]. This study reports the development of a rapid detection technique for Kenyan ToLCV isolates using probe-enhanced LAMP coupled with a modified APEG DNA extraction method in host crops. This screening methodology is crucial for monitoring the emergence and progression of pandemics caused by ToLCV in Kenya, facilitating the formulation of effective management plans to address and mitigate the prevalence of tomato-infecting viruses in affected regions and quarantine zones.

## 2 Materials and methods

### 2.1 Sample collection

A research permit for sample collection in the study counties (Baringo, Embu, Kajiado, Kirinyaga, Laikipia, and Narok) was granted by the National Commission for Science, Technology, and Innovation in April 2024 (NACOSTI/P/24/33998). A field survey was conducted from September to November 2024, and symptomatic and asymptomatic leaf samples suspected of being infected with leaf curl disease were collected. A total of 60 leaf samples consisting of 46 tomato and 14 chili pepper were collected from Baringo, Embu, Kajiado, Kirinyaga, Laikipia, and Narok counties in Kenya. Young leaves from symptomatic (exhibiting chlorosis/yellowing of the topmost young leaves, leaf size reduction, upward-curling leaf margins, stunting, and flower-drop) and asymptomatic tomato and chili pepper plants were gathered, stored in paper bags containing silica gel, transferred to the Pan African University Institute of Basic Sciences, Technology and Innovation (PAUISTI) Molecular Biology Laboratory, and stored at −80°C before further testing.

### 2.2 DNA extraction

#### 2.2.1 Modified CTAB combined with column purification.

Total genomic DNA was extracted using a modified CTAB method combined with column purification. Specifically, the initial steps of the CTAB method (tissue homogenization, lysis in CTAB buffer, incubation and phase clarification by centrifugation) were used for efficient disruption of plant tissues and removal of polysaccharides and secondary metabolites. This was followed by a column based purification step replacing conventional CTAB precipitation and organic extraction steps. The GeneAll® Exgene Plant SV mini kit served as a standard reference for DNA purification. DNA was isolated from the collected leaf samples of tomato and chili pepper obtained from the field utilizing a modified cetyltrimethylammonium chloride (CTAB) technique as described by Pratap *et al*. [45]. Briefly, approximately 100 mg of plant leaf material was crushed into a fine powder with liquid nitrogen utilizing a pestle and mortar and thereafter homogenized in 1 mL of CTAB extraction buffer (2% CTAB, 1% sodium metabisulfite, 1 g of PVP-40 (polyvinylpyrrolidone) in nuclease-free water, 100 mM Tris-HCl, 1.4 M sodium chloride, and 20 mM EDTA). The extraction buffer, to which 2% β-mercaptoethanol (v/v) was added immediately before use, was preheated to 85°C. The homogenized sap was placed into 1.5 mL microcentrifuge tubes, and incubation was performed at 65°C for 15 minutes, with shaking every 5 minutes to ensure material dispersion. Samples were centrifuged at 13,000 rpm (16,056 × g) (Hermle Z216M microcentrifuge equipped with a 24 × 1.5/2.0 mL fixed-angle rotor; Rotor Number: 220.87 V13) for 10 minutes at room temperature, and then 700 μl of the supernatant was aliquoted into fresh microcentrifuge tubes, followed by DNA column purification.

An equivalent volume of binding buffer BD was added into the supernatant and promptly vortexed for thorough mixing. Approximately 700 μL of the mixture was directly transferred into a GeneAll® SV spin column situated in a 2 mL collecting tube and spun for 30 seconds at 13 000 rpm (16,056 × g). The flow-through was disposed of, and the procedure was repeated with the residual sample. Subsequently, 700 μL of washing buffer CW was introduced into the spin column, centrifuged at 13,000 rpm (16,056 × g) for 30 seconds, and the effluent was discarded. An additional 300 μL of washing buffer CW was introduced into the spin column, centrifuged for 2 minutes at 13 000 rpm (16,056 × g), and the flow-through was discarded. The spin column was transferred to a fresh 1.5 mL microcentrifuge tube, to which 30 μL of elution buffer AE was directly introduced onto the center of the column membrane. Incubation was carried out at room temperature for 5 minutes, followed by centrifugation at 13 000 rpm (16,056 × g) for 1 minute. A Nanodrop (Genova Nano, Jenway) was utilized to determine the quantity and quality of the DNA (S3 File). Furthermore, DNA integrity was assessed on a 1% agarose gel. The obtained DNA was stored at −20°C and utilized for further analyses. The DNA which was extracted using the modified CTAB-column purification method was only used to prepare high quality reference (benchmark) DNA during assay development.

#### 2.2.2 Modified alkaline polyethylene glycol DNA extraction.

APEG buffer was prepared by mixing equal volumes of 20 mM NaOH (prepared by dissolving 0.8247 g NaOH in 1000 mL distilled water) and 6% (w/v) polyethylene glycol solution (formulated by dissolving 6 g of PEG in 100 mL of distilled water) (PEG 6000, Catalogue No. P8250, Solabio Life Sciences, Beijing Solarbio Science & Technology Co., Ltd.). The APEG buffer was modified by adding 1% (w/v) polyvinylpyrrolidone (PVP) and sodium chloride (NaCl) to give the following formulations:

1. APEG + NaCl was prepared by the addition of different molar concentrations of NaCl, 0.05 M, 0.1 M, 0.5 M, 0.8 M, and 1.0 M to APEG. The NaCl was added in its solid form.
2. APEG + PVP was prepared by adding 1% (w/v) polyvinylpyrrolidone. PVP was added to the APEG solution in its solid state.
3. APEG + PVP + NaCl was prepared by the addition of different molar concentrations of NaCl (0.05 M, 0.1 M, 0.5 M, 0.8 M, and 1.0 M) and 1% PVP. The NaCl and PVP were added in their solid state.

The extraction buffers were freshly prepared immediately before every use. APEG was used as the standard crude extraction method and its effectiveness was analysed along with the proposed modifications (APEG + NaCl, APEG + PVP, APEG + PVP + NaCl). The CTAB + GeneAll Exgene Plant SV Mini Kit served as the standard reference for DNA purification.

Approximately 50 mg of leaf tissue was suspended in 200 μl of newly formulated modified alkaline polyethylene glycol buffer in a 1.5 ml microcentrifuge tube and vortexed briefly. The tubes were centrifuged at 10,000 rpm (9,502 × g) for 30 seconds, followed by incubation at room temperature for 3 minutes to facilitate cell lysis. The lysate was then diluted 1:5 with nuclease-free water. The samples were subsequently maintained on ice until they were utilized. Then, 2 μl of the plant lysate was used for each reaction.

### 2.3 LAMP primer design

**Fig 1:**
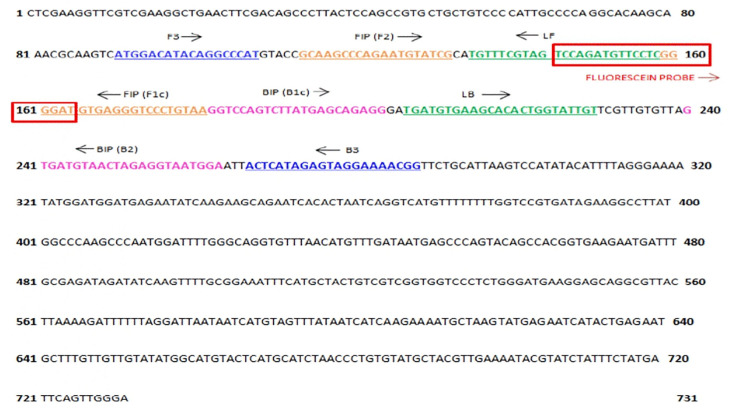
LAMP primers annealing and probe hybridization sites. F3 and B3 are depicted in blue; FIP (F1c-F2) is represented in orange; BIP (B1c-B2) is illustrated in violet and the two loop primers (LB and LF) are shown green. The arrows denote the direction of extension.

A primer set for the detection of the Kenyan ToLCV isolates using LAMP was developed using PrimerExplorer V5 software (https://primerexplorer.jp/e/) and the consensus nucleotide sequence of the AV1 coat protein gene as the amplification target. The consensus sequence was derived from a multiple sequence alignment of GenBank sequences of Kenyan ToLCV isolates, including MN894501.1, MN894504.1, MN894503.1, MN894493.1, MN894495.1, and MN894499.1 (S1 Fig). This ensured the captured viral diversity reflected circulating local strains, which is crucial for developing an effective regional diagnostic assay. The Kenyan ToLCV isolates analysed in this study are consistent with previously identified isolates from the region [3]. This high genetic similarity thus supports that these isolates represent the ToLCV populations currently found in Kenya. The primers used in the study comprised two loop-generating inner primers (loop forward and loop backward), the forward (F3) and backward (B3) primers, and the forward inner primer (FIP) and backward inner primer (BIP). Furthermore, a probe was manually designed by targeting the sequence located between the Fc3 and the Bc3 of the designated primer set (Table 1 and Fig 1).

**Table 1:** Primers used for Kenyan ToLCV isolates detection targeting the AV1 coat protein gene by LAMP.

Target gene	Primers	Primer sequence (5′ - 3′)	Primer position	Primer length (bp)
**AV1 coat protein gene**	F3	ATGGACATACAGGCCCAT	91–108	18
	B3	CCGTTTTCCTACTCTATGAGT	266–286	21
	FIP	TTACAGGGACCCTCACATCCC-GCAAGCCCAGAATGTATCG	114–180	40
	BIP	GGTCCAGTCTTATGAGCAGAGG- TCCATTACCTCTAGTTACATCAC	181–262	45
	LF	GAGGAACATCTGGACTACGAAACA	135–158	24
	LB	TGATGTGAAGCACACTGGTATTGT	205–228	24
	Probe	FITC-TCCAGATGTTCCTCGGGGAT	145–164	20

The probe was tagged with fluorescein isothiocyanate (FITC) to enable real-time detection of the amplicons through fluorescence. The primers and probes were subjected to BLAST analysis (https://blast.ncbi.nlm.nih.gov/blast.cgi) and assessed using the Sequence Manipulation Suite (https://www.bioinformatics.org/sms2/reference.html) to confirm specificity to ToLCV and the absence of significant homology with non-target sequences. All nucleotides and the probe were synthesized by GeneScript, Netherlands (Europe).

### 2.4 Polymerase chain reaction for detection of Kenyan ToLCV isolates

For PCR detection, both the modified CTAB + column purification and the modified APEG method were used to extract DNA from infected leaves of tomato and chili pepper plants, which served as templates. A 25 μL reaction was prepared comprising 12.5 μl Q5 High Fidelity 2× Master mix (New England Biolabs, UK), 2 μl template DNA, 1.0 μl each of 10 mM ToLCVF3 (5′ATGGACATACAGGCCCAT′3) primer and 10 mM ToLCVB3 (5′CCGTTTTCCTACTCTATGAGT′3) primer and 8.5 μl nuclease-free water. The ProFlex PCR thermocycler (Applied Biosystems, Life Technologies) was used for amplification using the following conditions: initial denaturation at 98°C for 30 seconds, followed by 35 cycles of denaturation at 98°C for 30 seconds, followed by annealing at 59°C for 45 seconds, extension at 72°C for 1 minute and 30 seconds, and final elongation at 72°C for 5 minutes. To detect ToLCV DNA, a PCR assay was conducted using primers targeting the AV1 coat protein region. The positive control was the *pMG-Amp* plasmid carrying the cloned AV1 coat protein gene, and the negative control was healthy plant DNA extract. Each reaction included a no-template control (NTC) with the complete reaction mixture, in which the template nucleic acid was replaced with nuclease free water. A VILBER E-BOX CX5.TS EDGE (France) UV gel documentation device visualized PCR amplicons on a 1% agarose gel. The expected band size was 220 bp, and samples that tested positive were used in downstream analysis.

### 2.5 Optimization of the real-time LAMP for detection of Kenyan ToLCV isolates

To conduct LAMP targeting the AV1 coat protein gene, six specific primers and a fluorescently labeled (FITC) probe were utilized. The LAMP reaction was initially performed with the *pMG-Amp* DNA plasmid carrying the cloned gene, followed by the use of DNA from infected tomato and chili pepper samples. The preliminary LAMP assay was performed in a 25 μl volume, containing 12.5 μl WarmStart® Fluorescent LAMP Master mix (New England Biolabs, US), 1.6 μM FIP/BIP primer, 0.2 μM F3/B3 primer, 0.4 μM LF/LB primer, 0.5 μl labeled probe, 2.5 μl LAMP fluorescent dye, and 2 μl template DNA. The volume was adjusted to 25 μl with nuclease-free water. The positive control was the *pMG-Amp* plasmid carrying the AV1 coat protein gene; the negative control was healthy plant DNA, and the no template control was nuclease-free water. These reactions were run in triplicate. The key factors considered in the optimization process were incubation temperature, reaction time, specificity of the primers used, method of DNA extraction, the inclusion or exclusion of the hybridization probe, and the presence or absence of loop primers. The LAMP reactions were conducted independently at 60°C, 63°C, and 65°C for 30–60 minutes. To determine the optimal amplification time, the LAMP reaction was conducted at 65°C with intervals of 30, 45, and 60 minutes.

The qTower384 (Analytik Jena) thermal cycler was used for real-time LAMP. Thermal cycling comprised amplification, inactivation, and melt curve analysis. Amplification was carried out at 65°C for 30 seconds per cycle and repeated for 60 cycles, followed by inactivation at 95°C for 2 minutes. The monitoring of LAMP amplification involved detecting fluorescence signals that surpassed a defined threshold readout. The analysis of the melt curve was performed by progressively raising the temperature from 60°C to 95°C, allowing for the identification of melting temperatures (Tm) of the amplified products. The cycle threshold and fluorescence intensity were plotted in amplification curves. The cycle threshold (Ct) was used to determine the time-to-positive (tp), defined as the time at which fluorescence reached the set relative fluorescence units (RFU) threshold. The tp value (minutes) was calculated from the cycle number, with each cycle lasting 30 seconds, using the following formula:


$$\begin{array}{c} tp(minutes)=\frac{Ct\times \text{3}\text{0}s(timepercycle)}{\text{6}\text{0}} \end{array}$$

Where:

*tp* – time-to-positive

*Ct* – Cycle threshold

All reactions were carried out in triplicate.

### 2.6 Optimization of colorimetric LAMP for detection of Kenyan ToLCV isolates

For colorimetric analysis, the WarmStart Colorimetric LAMP Master Mix (New England Biolabs, US) was used with LAMP primers (outer, inner, and loop) at concentrations of 0.2 μM, 1.6 μM, and 0.4 μM, respectively, along with 0.5 μL of the probe and 2 μL of template DNA. The *pMG-Amp* plasmid carrying the AV1 coat protein gene served as the positive control, healthy plant DNA as the negative control, and nuclease-free water as the no template control. End-point detection was conducted in a water bath. The experimental process was composed of amplification at 65°C for 30 minutes and inactivation at 80–90°C for 2 minutes. This step was also carried out only once. Positive reactions were represented by a color change from pink to yellow, while negative reactions retained the pink hue. All reactions were performed in triplicate. After amplification, the samples were diluted 5-fold by adding 4 μL of the LAMP reaction sample to 16 μL of nuclease-free water, and separation was performed on a 2% agarose gel.

### 2.7 Determination of the sensitivity and specificity of the LAMP assay

To determine the sensitivity of the LAMP assay, a series of ten-fold dilutions ranging from 10^−1^ to 10^−12^ of total DNA extracted from a ToLCV- infected leaf sample that had previously tested positive by PCR, was prepared. Subsequently, 2 μL of each dilution was used as templates for both LAMP and PCR assays (as described in Section 2.4 and Section 2.5). The detection limits were established by the minimum input DNA concentration that yielded a positive result. The specificity of the LAMP assay and the assessment of potential non-specific cross-reactions with other tomato-infecting viruses were conducted using DNA prepared from two samples infected with ToLCV together with cDNA from tomato spotted wilt virus (TSWV), impatiens necrotic spot virus (INSV), potato virus Y (PVY), tobacco mosaic virus (TMV), tomato mosaic virus (ToMV), and cucumber mosaic virus (CMV). In each run, total DNA extracted from a healthy tomato plant served as the negative control, the cloned *pMG-Amp* plasmid as the positive control, and nuclease-free water was used as the no-template control. Each sample was analyzed in triplicate.

### 2.8 Evaluation of optimized LAMP assay

The LAMP assay was first evaluated in the laboratory using the *pMG-Amp* plasmid DNA. An evaluation of the assay was conducted using 60 infected leaf samples collected from tomato and chili pepper fields exhibiting typical ToLCV symptoms, as well as healthy plants. A total of 8 asymptomatic plant samples were utilized in the validation process. To validate the LAMP findings, total DNA was isolated from the field-collected leaf samples using a modified APEG method (S3 File), and then it was amplified by LAMP using the Kenyan ToLCV isolates-specific primers and probe. Real time quantitative PCR (qPCR) was carried out to evaluate samples which tested negative for conventional PCR but tested positive for real time LAMP. Total DNA was extracted from the leaf samples using the modified APEG method (S4 File and S2 Fig). A 10 μL reaction mix comprising 5 μL Luna Universal 2× qPCR master mix (New England Biolabs, US), 1 μL (50 ng/μL) of template DNA, 0.5 μL each of 10 mM ToLCVF3 (5′ATGGACATACAGGCCCAT′3) primer and 10 mM ToLCVB3 (5′CCGTTTTCCTACTCTATGAGT′3) primer and 2 μL nuclease free water was constituted. The qTower384 (Analytik Jena, Endress+Hauser Company) was used for amplification using the following thermal profile: initial denaturation at 95°C for 2 minutes, followed by 40 cycles of denaturation at 95°C for 15 seconds and annealing and extension at 60°C for 60 seconds. The positive control was DNA extracted from a biologically positive sample, the negative control was healthy plant DNA and the no template control included nuclease free water. These reactions were run in triplicate.

### 2.9 Data analysis and diagnostic performance evaluation

The data collected were analyzed using qTower384 v1.0 software (AnalytikJena, Endress+Hauser Company), and GraphPad Prism v10.6.1 software was used for statistical analysis. Dunnett’s multiple comparison test was used to evaluate the differences between groups after a one-way ANOVA. The Dunnett’s test was chosen to control the family-wise error rate when comparing multiple treatments to a single control, and it offers higher statistical power than more conservative methods. Data from the LAMP reaction in the presence and absence of loop primers were analyzed using the two-tailed, unpaired t-test. Statistical significance was defined as p < 0.05. All experiments were run in triplicate.

The diagnostic accuracy of the LAMP assay and conventional PCR was assessed using a 2 × 2 contingency table analysis. True positives (TP), true negatives (TN), false positives (FP), and false negatives (FN) were enumerated. Sensitivity (proportion of infected samples correctly detected), specificity (proportion of uninfected samples correctly excluded), positive predictive value (PPV), and negative predictive value (NPV) were calculated as follows: sensitivity = TP ÷ (TP + FN); specificity = TN ÷ (TN + FP); PPV = TP÷ (TP + FP); NPV = TN ÷ (TN + FN). Positive and negative likelihood ratios, LR^+^ = sensitivity ÷ (1 − specificity) and LR^−^ = (1 − sensitivity) ÷ specificity, evaluated the test’s capacity to distinguish infection. Ninety-five percent confidence intervals (95% CIs) for sensitivity, specificity, predictive values, and likelihood ratios were estimated using the Wilson method [46] for reliable intervals. Analyses were conducted with GraphPad version 10.6.1 (GraphPad Software Inc., La Jolla, CA, USA). Agreement between assays was assessed using Cohen’s kappa coefficient (κ), calculated using the *irr* package in R v4.5.3, and interpreted according to established benchmarks. Receiver operating characteristic (ROC) analysis compared the binary LAMP assay against PCR classification results [47] by plotting sensitivity versus (1 − specificity) and calculating the area under the curve (AUC) with 95% confidence intervals using DeLong’s method in the *pROC* package for R v4.5.3 (RStudio-2026.01.1-403). While ROC analysis is typically applied to continuous variables, it can be applied to binary classification (positive vs negative) to yield a valid metric of diagnostic accuracy when combined with sensitivity and specificity. For binary tests, ROC analysis yields a single operating point, with the AUC equal to the arithmetic mean of sensitivity and specificity. All analyses applied a statistical significance threshold of p < 0.05.

## 3 Results

### 3.1 Detection of Kenyan ToLCV isolates using LAMP

**Fig 2:**
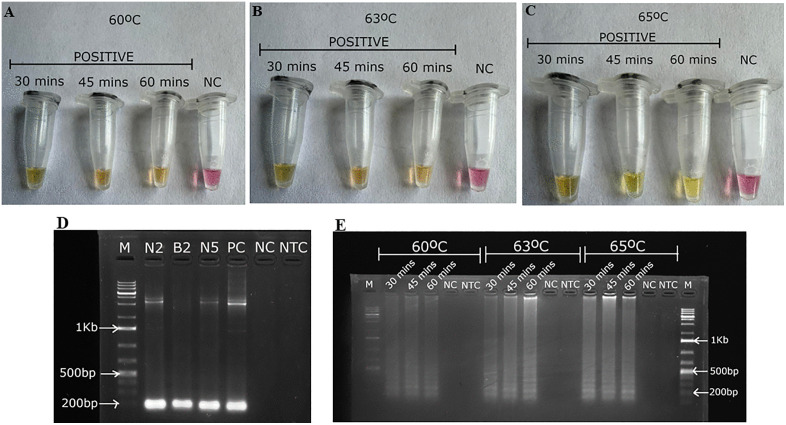
The detection of Kenyan ToLCV isolates using LAMP and conventional PCR. **(A)**, **(B)** and **(C)** Visual inspection after LAMP: yellow – positive samples, pink – negative samples at 60°C, 63°C and 65°C respectively; **(D)** Visualization of PCR product after gel electrophoresis at 95mV for 1 hour on a 1% gel. Lanes B2, N2 and N5 represent positive samples. **(E)** Visualization of LAMP products after gel electrophoresis at 95 mV for 1 hour on a 2% gel. Lane M – 1Kb DNA ladder (O’GeneRuler Plus 1Kb ladder, Thermo Scientific), PC – positive control, NC – negative control, NTC – no template control.

Initial optimization was done for the detection of Kenyan ToLCV isolates using the *pMG-Amp* plasmid carrying the AV1 gene and DNA extracted from selected confirmed positive samples from the field which had been naturally infected with the virus. The LAMP assay was carried out at 60°C, 63°C and 65°C. The optimal temperature for isothermal amplification was found to be 65°C (Figs 2A–2C), and was used for all successive LAMP reactions.

Optimal amplification occurred at 65°C, at which the characteristic pink-to-yellow color transition indicative of a positive reaction was most clearly observed. Amplification was also observed at 60°C and 63°C; however, the color change for the colorimetric assay from pink to orange was observed (Figs 2A–2C) indicating that amplification was incomplete. Healthy plant DNA was included as a negative control (NC) and nuclease free water as the no template control to assess the efficacy of the primer set developed for Kenyan ToLCV isolates detection and also to check for any cross over contamination. The findings indicated that specific amplification products were produced during amplification of all the infected samples and in reactions using the *pMG-Amp* plasmid positive control. The LAMP reaction was also run at 30, 45 and 60 minute intervals to determine the optimal amplification time. The optimal amplification time was found to be 30 minutes and was adopted for all successive LAMP reactions (Figs 2A–2C).

In contrast, no amplification products were observed in uninfected control samples. Positive reactions exhibited a transition in color from pink to yellow, whilst there was no color change for negative reactions. The presence of the ladder-like band pattern on the gel (Fig 2E) also confirmed positive LAMP reactions. This ladder-like pattern is characteristic of LAMP amplification, resulting from *Bst* polymerase-mediated strand displacement using six primers targeting multiple sites on the template. Conventional PCR detection of the Kenyan isolates of ToLCV produced bands with a size of ~220 bp on the gel (Fig 2D).

**Fig 3:**
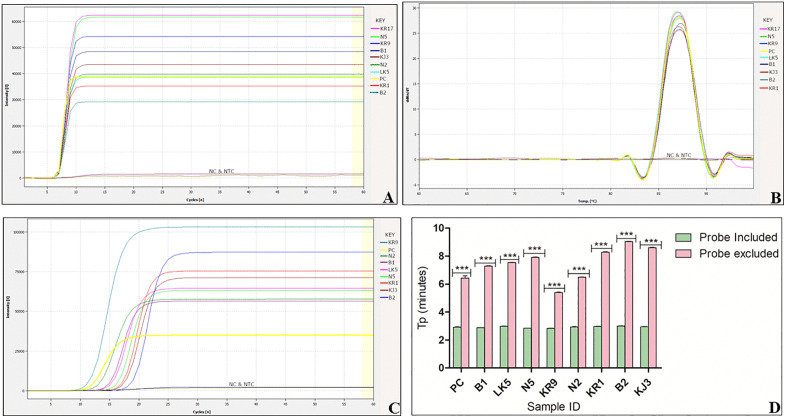
Amplification plot for real time detection of Kenyan ToLCV isolates using LAMP including and excluding the hybridization probe. **(A)** The amplification curve for LAMP reaction (including the hybridization probe) showing Ct (cycle threshold) values of ~5.5 to 6.5 which translate to a time to positive (tp) of ~2.7 to 3.5 minutes **(B)** Melt curve profile of ToLCV amplicons. PC – positive control, NC – negative control and NTC – no-template control. **(C)** Amplification curve for LAMP reaction (excluding the detector probe) showing Ct values of ~10.8 to 18.2 which translate to a time to positive (tp) of ~ 5.4 to 9.1 minutes. **(D)** Graph plot showing time to positive (Tp) for reactions including and excluding the detector probe (***p < 0.0001).

The incorporation of a fluorescent dye into the reaction mix enabled the generation of real-time data sets. The fluorescence data were plotted in relation to the cycle threshold (Ct), and Fig 3A demonstrates the anticipated sigmoidal curve for each positive sample, which includes the *pMG-Amp* plasmid DNA (positive control) as well as the infected samples collected from the field.

Furthermore, the amplification product of each sample was observed in 2.7 to 3.5 minutes (threshold time). No amplification was detected in the negative controls even after extended reaction times, as anticipated. The melt curves of the positive LAMP reactions showed a consistent temperature of approximately 86°C (Fig 3B). The data are presented as means ± SD of three replicates, with analysis conducted using the t-test (S6 File). The tp value ranges of 2.7 to 3.5 minutes and 5.4 to 9.1 minutes for reactions with and without the detector probe, respectively (Figs 3C and 3D), demonstrate that the inclusion of the detector probe enhances the speed of the reaction.

**Fig 4:**
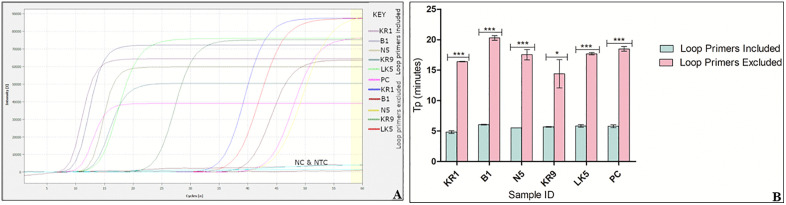
Effect of presence or absence of loop primers on LAMP reaction. **(A)** Amplification curves showing reaction including loop primers and excluding loop primers. PC is the positive control, NC is the negative control and NTC is the no- template control. **(B)** Graph plot showing time to positive (Tp) for reactions including and excluding loop primers (*** p < 0.0001 and *p = 0.0199).

Furthermore, the LAMP reaction was conducted both with and without the loop primers (Fig 4).

The data are presented as means ± SD of three replicates, with analysis conducted using the t-test (S6 File). The tp values of 4.3–6.2 minutes and 15.1–20.2 minutes for reactions with and without loop primers, respectively (Figs 4A and 4B), demonstrate that the inclusion of loop primers enhances the speed of the reaction.

### 3.2 Optimization of the APEG DNA extraction method

**Fig 5:**
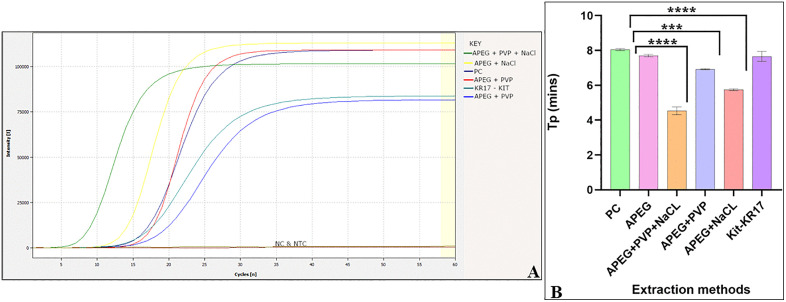
Effect of different extraction methods on LAMP reaction. **(A)** Amplification curves and **(B)** graph plot showing time to positive (Tp) for different extraction methods. 1 – APEG+PVP + NaCL, 2 – APEG+NaCL, 3 – APEG+PVP, 4 – APEG and KR17 – KIT. PC – positive control, NC – negative control and NTC – no template control (***p = 0.0007, ****p < 0.0001).

LAMP was performed using DNA templates from different extraction methods, including commercial kit-extracted DNA (CTAB + column purification), APEG-extracted DNA, and modified APEG-extracted DNA (Fig 5).

The data are expressed as means ± SD of 3 replicates. No significant variation in time to positivity was observed in LAMP amplification when DNA was extracted using the APEG method (p > 0.05) (S6 File). The APEG extraction method demonstrated practical suitability for rapid applications, with an extraction time of ~5 minutes and has potential compatibility for field analysis, as reagents can be transported and stored at room temperature. Further modifications to the APEG extraction method all resulted in improved efficiency of LAMP amplification. The inclusion of PVP and NaCl in the extraction buffer produced a statistically significant improvement in amplification efficiency (p < 0.0001) (Fig 5A and 5B). Reduced time-to-positive is indicative of improved reaction kinetics and it is associated with higher template quality and lower inhibitor presence in isothermal amplification assays. The optimized APEG + PVP + NaCl formulation yielded the shortest reaction time, was identified as the most effective extraction approach, and was adopted for all subsequent reactions.

**Fig 6:**
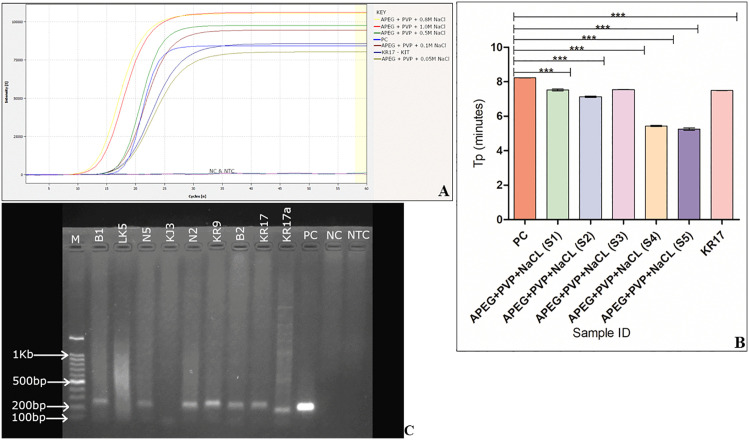
Effect of different NaCL concentrations in extraction buffer on LAMP reaction. **(A)** Amplification curves and **(B)** graph plot showing the time to positive (Tp) at different concentrations of the NaCL. S1 - 0.05 M, S2 - 0.5M, S3 - 0.1 M, S4 - 1.0 M and S5 - 0.8 M (***p < 0.0001). **(C)** Visualization of PCR product from DNA extracted using the modified APEG+PVP + 0.8 M NaCL after gel electrophoresis at 95mV for 1 hour on a 1% gel. Lane B1 to KR17 – DNA extracted using the modified APEG+PVP + 0.8 M NaCL; KR17a - commercial kit extracted, Lane M – 100 bp DNA ladder (Smartcheck, Accuris), PC – positive control, NC – negative control, NTC – no template control.

The APEG+PVP + NaCl DNA extraction method was further optimized at different NaCl concentrations (Fig 6).

The data are presented as means ± SD of 3 replicates. Increasing the NaCl concentration from 0.05 M to 1.0 M generally enhanced the efficiency of LAMP, as indicated by the lower amplification times (tp). A significant variation in amplification time was observed for S5 (NaCl concentration of 0.8 M) (p < 0.0001). The modified buffer with an NaCl concentration of 0.8 M had the shortest amplification time (tp) of ~4.8 minutes (Fig 6B) and was adopted for the modified alkaline lysis buffer protocol. An analysis of the gel for the PCR product (Fig 6C) from DNA extracted using the modified APEG method, which contains 0.8 M NaCl, shows that the technique produces high-quality DNA. The modified APEG + PVP + 0.8 M NaCl buffer was adopted for all subsequent reactions.

### 3.3 Analytical sensitivity of the probe-enhanced LAMP assay

**Fig 7:**
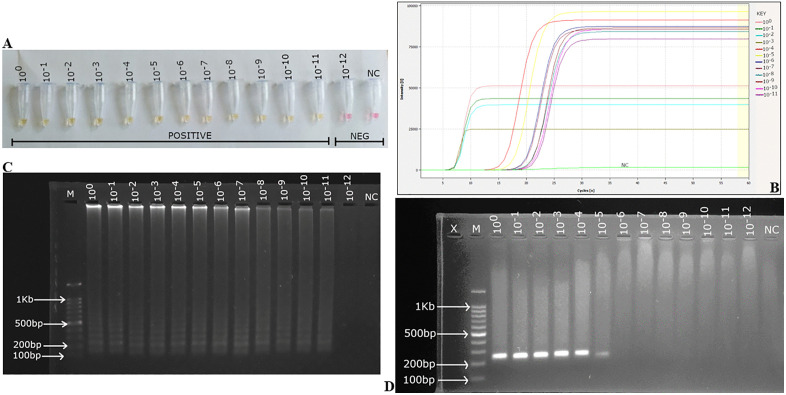
Sensitivity of the conventional PCR and real time LAMP assays for Kenyan ToLCV isolates detection using 10-fold serial dilution. **(A)** Colorimetric observation for LAMP serial dilution. **(B)** Amplification curves showing fluorescence increasing (from 10^−1^ to 10^−11^ serial dilutions) after 3.4 minutes to 9.9 minutes. **(C)** Limit of detection was determined to be ~ 100 × 10^−11^ ng/μL (0.001 fg/μL) using agarose gel electrophoresis. **(D)** Agarose gel electrophoresis of PCR amplicons showing limit of detection to be ~100 × 10^−5^ ng/μL (0.001 ng/μL). M: 100 bp ladder marker: NTC: No template control.

The analytical sensitivity of the assay was determined through real-time detection and gel electrophoresis, utilizing various concentrations of template DNA, starting at 100 ng/μL and employing 10-fold serial dilutions. The DNA template, serially diluted from a concentration of ~100 ng/μL (10^0^) to 0.0001 fg/μL (10^−12^), exhibited an amplification characterized by a color change from pink to yellow, the appearance of the multiple ladder-like band pattern on the gel and a time to positive from 3.4 minutes to 9.9 minutes (Fig 7).

The LAMP assay (Figs 7A–7C) detected the virus up to a dilution of DNA of ~100 × 10^−11^ ng/μL, but the PCR failed to detect beyond ~100 × 10^−5^ ng/μL (Fig 7D). The most concentrated sample, 10^0^, had the lowest time to amplification at 3.4 minutes (Fig 7B). The sensitivity assay indicated a high degree of analytical sensitivity of the LAMP assay.

### 3.4 Specificity analysis results

**Fig 8:**
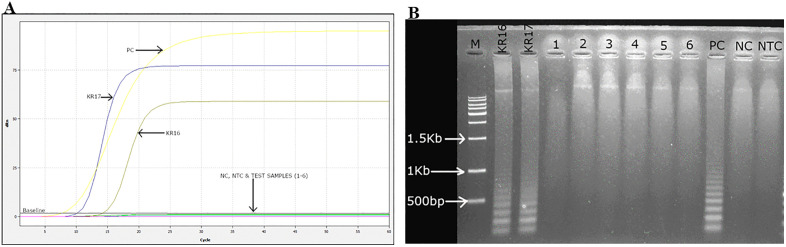
Determination of specificity of LAMP for detection of Kenyan ToLCV isolates. **(A)** Amplification curves showing ToLCV positive KR16 and KR17. **(B)** Agarose gel electrophoresis of LAMP amplicons. 1 – TSWV, 2 – INSV, 3 – PVY, 4 – TMV, 5 – ToMV and 6 – CMV, M – 1Kb molecular ladder (New England Biolabs, US), N – 100 bp molecular ladder (Bioline), PC – positive control, NC – negative control and NTC – no template control.

The specificity of the LAMP assay was evaluated, and potential specific cross-reactions with other tomato-infecting viruses were assessed through a LAMP assay involving six viruses described in Section 2.7. The results (Fig 8) of the optimized LAMP assay analyzed by gel electrophoresis showed successful amplification of the AV1 gene of the Kenyan ToLCV isolates from DNA extracted from infected samples.

No amplification was detected in non-target viruses, healthy samples, and no-template controls (Figs 8A and 8B), as indicated by the absence of a ladder-like band pattern on gel electrophoresis for any of the tomato-infecting viruses. The results depicted in Fig 8 indicate that the primers and probes utilized in this study exhibited high specificity, as amplification and detection were exclusively observed in samples containing ToLCV. The findings validate the specificity of the assay and exclude cross-reactivity with other viruses that infect tomatoes.

### 3.5 Assay evaluation of infected field samples

**Fig 9:**
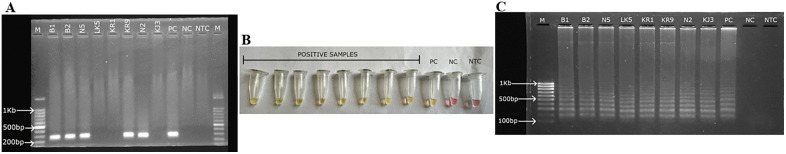
The detection of Kenyan ToLCV isolates from different field samples. **(A)** Visualization of PCR product after gel electrophoresis at 95mV for 1 hour on a 1% gel. Lanes B1, B2 and N5 represent – symptomatic tomato, while Lanes LK5, KR1 and KJ3 represent – asymptomatic tomato plants; Lane N2 represents – symptomatic chili pepper plant. **(B)** Visual inspection after LAMP: yellow – positive samples, pink – negative samples and **(C)** Visualization of LAMP products after gel electrophoresis at 95 mV for 1 hour on a 2% gel. Lane M – 100 bp DNA ladder (Bioline), PC – positive control, NC – negative control, NTC – no template control.

To further validate the results obtained, a total of 60 samples, comprising 46 tomato and 14 chili pepper leaf samples, collected from infected symptomatic and asymptomatic tomato and chili pepper plants from the field were screened for ToLCV using PCR (as detailed in Section 2.4), with positive samples characterized by an anticipated band size of approximately 220 bp on the gel (Fig 9A).

The asymptomatic plants included samples presumed to be healthy and virus-free. The same DNA samples were subjected to real-time and colorimetric loop-mediated isothermal amplification (Figs 9B and 9C). Results show that LAMP was able to detect Kenyan isolates of ToLCV in asymptomatic samples whereas conventional PCR could not.

Out of the 60 samples, 22 were positive, and 38 were negative by PCR, whilst 26 out of 60 were ToLCV positive by LAMP assay (Table 2).

**Table 2:** Comparison of LAMP and PCR coupled with modified APEG extraction method for detection of ToLCV from field samples.

Sample code	Sampled vegetable crop	Symptomatology	Modified APEG + PCR	Modified APEG + LAMP
KR1	Tomato	Asymptomatic	**−**	**+**
KR2	Tomato	Symptomatic	**−**	**−**
KR3	Tomato	Symptomatic	**−**	**−**
KR4	Tomato	Symptomatic	**−**	**−**
KR5	Chili pepper	Symptomatic	**−**	**−**
KR6	Tomato	Symptomatic	**−**	**−**
KR7	Tomato	Symptomatic	**−**	**−**
KR8	Tomato	Asymptomatic	**−**	**−**
KR9	Tomato	Symptomatic	**+**	**+**
KR10	Chili pepper	Asymptomatic	**−**	**−**
KR11	Tomato	Symptomatic	**−**	**−**
KR12	Tomato	Symptomatic	**−**	**−**
KR13	Tomato	Symptomatic	**−**	**−**
KR14	Tomato	Symptomatic	**−**	**−**
KR15	Tomato	Symptomatic	**−**	**−**
KR16	Tomato	Symptomatic	**+**	**+**
KR17	Tomato	Symptomatic	**+**	**+**
KR18	Tomato	Symptomatic	**+**	**+**
KR19	Tomato	Symptomatic	**+**	**+**
KR20	Tomato	Symptomatic	**+**	**+**
KR21	Tomato	Symptomatic	**−**	**−**
KR22	Chili pepper	Symptomatic	**+**	**+**
KR23	Chili pepper	Symptomatic	**−**	**−**
KR24	Tomato	Symptomatic	**−**	**−**
LK1	Tomato	Symptomatic	**−**	**−**
LK2	Tomato	Symptomatic	**+**	**+**
LK3	Tomato	Symptomatic	**−**	**−**
LK4	Chili pepper	Symptomatic	**+**	**+**
LK5	Tomato	Asymptomatic	**−**	**+**
LK6	Chili pepper	Symptomatic	**+**	**+**
LK7	Chili pepper	Symptomatic	**+**	**+**
LK8	Chili pepper	Symptomatic	**+**	**+**
LK9	Tomato	Symptomatic	**−**	**−**
LK10	Tomato	Symptomatic	**+**	**+**
LK11	Tomato	Asymptomatic	**−**	**−**
KJ1	Tomato	Symptomatic	**−**	**−**
KJ2	Chili pepper	Symptomatic	**+**	**+**
KJ3	Tomato	Asymptomatic	**−**	**+**
KJ4	Tomato	Symptomatic	**−**	**−**
KJ5	Tomato	Symptomatic	**−**	**−**
KJ6	Tomato	Asymptomatic	**−**	**+**
KJ7	Tomato	Symptomatic	**−**	**−**
KJ8	Tomato	Symptomatic	**−**	**−**
KJ9	Chili pepper	Symptomatic	**+**	**+**
KJ10	Tomato	Asymptomatic	**−**	**−**
KJ11	Tomato	Symptomatic	**−**	**−**
KJ12	Tomato	Symptomatic	**−**	**−**
KJ13	Chili pepper	Symptomatic	**+**	**+**
KJ14	Tomato	Symptomatic	**−**	**−**
KJ15	Tomato	Symptomatic	**−**	**−**
B1	Tomato	Symptomatic	**+**	**+**
B2	Tomato	Symptomatic	**+**	**+**
B3	Tomato	Symptomatic	**−**	**−**
B4	Tomato	Symptomatic	**+**	**+**
B5	Tomato	Symptomatic	**+**	**+**
N1	Tomato	Symptomatic	**−**	**−**
N2	Chili pepper	Symptomatic	**+**	**+**
N3	Chili pepper	Symptomatic	**−**	**−**
N4	Chili pepper	Symptomatic	**−**	**−**
N5	Tomato	Symptomatic	**+**	**+**

KR – Kirinyaga; LK – Laikipia; KJ – Kajiado; B – Baringo; N – Narok.

Furthermore, 8 out of 60 were asymptomatic based on visual detection. All field sampling was conducted with an experienced agronomist from the Kenyan Plant Health Inspectorate Service, following standard phytosanitary protocols. The sampled plants showed no visible ToLCV symptoms, such as stunting, leaf curling, or flower abscission. Sampling occurred during the active vegetative stage when symptoms are typically evident. All the asymptomatic samples were from the Ansal F1 hybrid cultivar, a commercially marketed variety reported to possess resistance to ToLCV. Hybrid tomatoes often carry resistance genes that limit viral replication or delay symptom onset. Furthermore, changes in host-virus interactions in hybrids may slow the spread of infection or reduce symptom severity [3]. The 8 asymptomatic samples also tested negative by conventional PCR. The LAMP assay successfully detected Kenyan ToLCV isolates in 50% (4 out of 8) of the asymptomatic samples. These results show that the probe-enhanced LAMP is more efficient for detection of Kenyan ToLCV isolates, even at low viral titers in asymptomatic samples.

### 3.6 Evaluation of asymptomatic samples using real time qPCR

**Fig 10:**
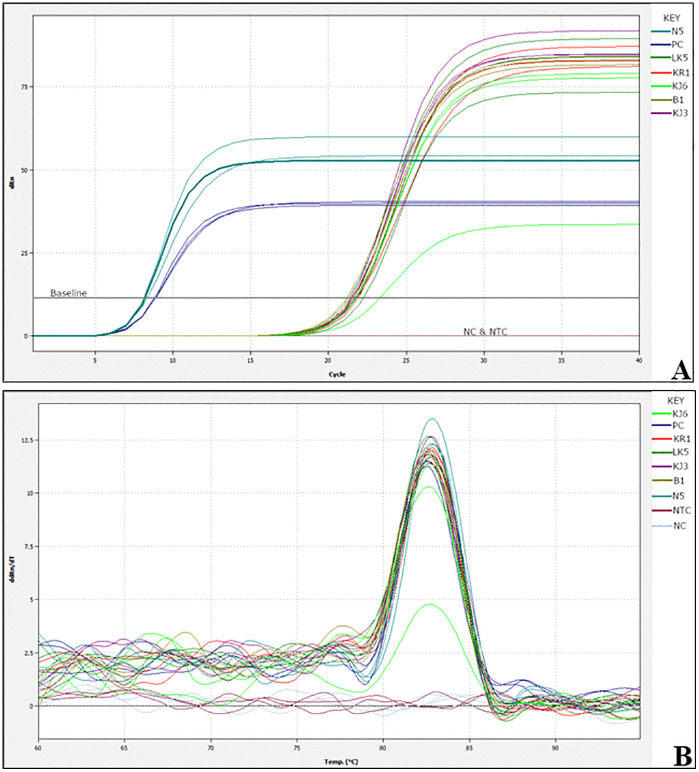
Real time qPCR assay for detection of Kenyan ToLCV isolates in asymptomatic samples. **(A)** Amplification curves for real time qPCR reaction showing mean Ct values ranging from 21.41 ± 0.09 to 22.21 ± 0.3 minutes **(B)** Melt curve profile of the ToLCV amplicons. PC – positive control, NC – negative control and NTC – no template control.

The probe enhanced LAMP assay was able to detect Kenyan ToLCV isolates in four (4) asymptomatic leaf samples that tested negative by conventional PCR. Evaluation using real-time qPCR was done in order to verify that the positivity was due to true amplification and not due to cross contamination or false positives. All the four asymptomatic samples tested positive by real time qPCR. Mean Ct values ranged from 21.41 minutes ± 0.09 to 22.21 minutes ± 0.3 (Fig 10A), based on 1-minute cycle duration, indicating low viral loads consistent with LAMP detection at the detection threshold.

Analysis of the melt curves of the positive reactions showed a mean consistent temperature of approximately 82.67°C ± 0.09 (Figs 10B and S3). No amplification was observed in the negative and no template controls, indicating that amplification was due to the presence of infection by ToLCV and not due to contamination.

### 3.7 Diagnostic performance of the probe enhanced LAMP assay

The probe-enhanced LAMP assay exhibited high diagnostic performance in the tested samples (Table 3 and S7 File).

**Table 3:** Diagnostic performance metrics.

Parameter	Value	95% Cl
Sensitivity	1.000	0.851–1
Specificity	0.895	0.759–0.958
Positive Predictive Value	0.846	0.665–0.938
Negative Predictive Value	1.000	0.898–1
Accuracy	0.933	0.841–0.974
Cohen’s kappa	0.862	0.713–0.967
ROC AUC	0.947	0.898–0.997 (DeLong)

**Fig 11:**
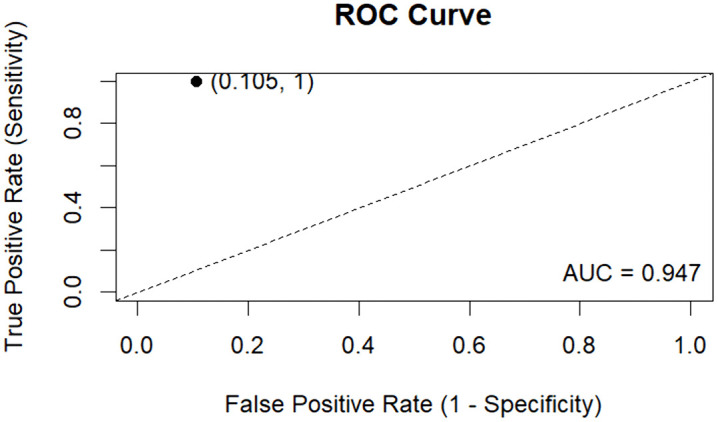
Receiver operating characteristic (ROC) analysis of the LAMP assay for detection of Kenyan ToLCV isolates.

The sensitivity observed confirmed that all positive samples were accurately detected. The specificity was 0.895 (95% CI: 0.759–0.958), indicating a small proportion of discordant positive results relative to the reference method. High PPV and NPV demonstrate that negative results were consistently concordant with the reference standard in this dataset. The overall accuracy was 0.933 (95% CI: 0.841–0.974). The assay demonstrated strong concordance with the reference method, as evidenced by a high Cohen’s kappa value which was statistically significant (Z = 6.74, p = 1.59 × 10^−11^). The positive likelihood ratio (LR+) was 9.5, indicating that a positive LAMP result was 9.5 times more likely in infected samples relative to uninfected ones. The negative likelihood ratio (LR−) was 0.000, indicating the absence of false negatives. The area under the ROC curve (AUC) quantitatively assesses the diagnostic performance; the observed AUC was 0.947 (95% CI: 0.898–0.997), signifying robust discriminative capability. The ROC curve corresponds to a single operating point (false positive rate = 0.105, true positive rate = 1.000), reflecting the observed sensitivity and specificity (Fig 11).

## 4 Discussion

Rapid on-site screening of infected plants can significantly reduce considerable financial losses, especially for smallholder farmers whose livelihoods depend on horticultural produce. While identifying causative agents is essential for effective disease management, relying solely on visual assessment of disease indicators presents significant challenges. This is because the symptoms induced by most plant viruses are frequently indistinguishable by visual assessment alone. Therefore, it is essential to develop precise detection techniques for each virus. Due to the complexity of these viruses and their evolving epidemiology, characterized by emergent strains, pandemic spread, and host adaptation, disease management is rendered considerably more challenging. Horticultural crops, such as tomato and chili pepper, are under constant threat from plant viral pathogens, emphasizing the necessity for effective management strategies. To prevent excessive pesticide application and effectively manage the insect vector (whitefly, *B. tabaci*), the best strategy for preventing viral spread is Integrated Pest Management (IPM), where early plant pathogen diagnosis plays a crucial role. Thus, the use of sensitive and accurate diagnostic and detection tools is crucial for effective disease management and preventing the spread of viral diseases.

This study employed the LAMP technique to develop an efficient, swift method for detecting Kenyan tomato leaf curl virus isolates in both asymptomatic and symptomatic plants, coupled with a rapid, modified APEG-based DNA extraction method suitable for field conditions. To improve the efficiency of the LAMP assay for on-site diagnostics, particularly in resource-limited environments, it is crucial to create a method that requires minimal instrumentation. The LAMP primers developed for this study successfully amplified the target AV1 gene at 65°C within 30 minutes. The incorporation of loop primers (Fig 4) significantly accelerated the LAMP reaction, improving its sensitivity and efficiency while reducing the reaction time by approximately 50% [27,48]. The inclusion of loop primers enhances the number of initiation sites in the LAMP reaction, as supported by earlier research [20,27]. A single-step colorimetric method was optimized, producing a color change readily detectable by the naked eye. In previous studies, colorimetric tests for virus detection have been optimized by adding different dyes [35]. The results of this study are consistent with earlier reports that demonstrate a comparable color change during isothermal amplification [49].

This study focuses on the AV1 coat protein region as the target for LAMP design (Table 1 and Fig 1), chosen for its low recombination rate. The AV1 gene shows lower variability across ToLCV isolates than other viral genes [3]. This enhances its reliability as a target for amplification and detection [3,33]. The primers ToLCVF3/B3 successfully amplified a 220 bp ToLCV DNA sequence (Fig 2E) using PCR. The optimal conditions for LAMP in detecting Kenyan isolates of ToLCV were established at 65°C within 30 minutes, as supported by previous research [27]. This temperature is optimal for the activity of *Bst* DNA polymerase, which plays a vital role in the reaction [50,51]. The probe-enhanced LAMP assay detected Kenyan ToLCV isolates in infected samples within 2.7 to 3.5 minutes (Fig 3A), whereas the conventional PCR assay requires at least 3 hours for virus detection, including the gel electrophoresis step. Moreover, LAMP is faster and simpler than traditional PCR, enabling higher throughput without additional resources [52]. A recommendation was made that for a point-of-care testing method to be considered for pathogen detection, it should have a turnaround time of less than 30 minutes [53]. In this study, the probe-enhanced LAMP assay detected the ToLCV within 30 minutes, meeting the requirements for point-of-care testing (POCT). The analysis can be performed using a water bath, thereby eliminating the need for electricity. Furthermore, the ease of sample preparation and the availability of a lyophilized LAMP reaction mixture eliminated the requirement for skilled operation or specialized apparatus.

The limit of detection of the developed test was determined by performing LAMP with a series of diluted samples, initially set at approximately 100 ng/μL. This study achieved an analytical sensitivity of 100 × 10^−11^ ng/μL (0.001 fg/μL). This detection limit compares favorably with or exceeds those reported for other LAMP-based diagnostics for tomato leaf curl virus and related begomoviruses. Most published ToLCV LAMP assays report detection limits of approximately 10^−6^ to 10^−9^ ng/μL, depending on the test setup, primers, and detection method [29,32,33,44]. Although the probe-enhanced LAMP assay demonstrated a ~10⁶-fold increase in sensitivity over conventional PCR, this substantial enhancement is method-specific, resulting from probe incorporation, primer optimization, and optimized reaction conditions rather than a universal property of LAMP. Importantly, the implications of this enhanced sensitivity are particularly relevant for begomovirus epidemiology. ToLCV infections are known to occur at low viral titers during early infection stages or in asymptomatic hosts, which often act as reservoirs for disease spread. The ability to detect viral nucleic acids at low concentrations, therefore, represents a meaningful advancement for early detection and surveillance. This assay demonstrates sensitivity comparable to quantitative PCR, is isothermal, and requires a significantly shorter reaction time. The superior detection limits, rapidity, and simplicity of the assay make probe-enhanced LAMP an effective tool for point-of-care diagnosis of ToLCV in Kenya.

The Kenyan ToLCV isolates LAMP assay established in this study exhibited high specificity, as no amplification products were detected in samples infected with other tomato-infecting viruses, including TSWV, ToMV, TMV, INSV, PVY, and CMV. These results concur with prior research findings [33,51]. These six common non-target tomato viruses co-circulate on Kenyan farms [51], reflecting real diagnostic conditions. These viruses are most likely to cause cross-reactivity and thus provide a realistic test of the assay’s specificity. The high specificity of this assay, confirmed by the absence of cross-reactivity with the six common tomato-infecting viruses, and its high sensitivity are complementary properties that together minimize both false-positive and false-negative rates in field diagnostics. The frequent occurrence of false positives due to non-specific amplification can be attributed to primer-dimer interactions. Primer-dimer artefacts, promoted by the high primer concentrations inherent to LAMP, can occasionally generate false-positive signals. According to the cross-amplification test results, the developed assay exhibits a low risk of false positives and false negatives. The design of a unique set of six primers, incorporating loop primers and a specific hybridization probe, effectively minimized false-positive signals, thereby enhancing detection efficiency and specificity. The primers were developed utilizing the heterologous region of the gene sequence. Furthermore, implementing a one-pot reaction setup effectively reduced carryover contamination.

Early LAMP assays demonstrated sensitivity 10–100 times higher than conventional PCR [35], but lacked the ability to confirm sequence specificity. Newer approaches, such as real-time LAMP and improved sample preparation, have made field deployment easier [33]. In this study, the probe-integrated LAMP method stands out for its use of sequence-specific hybridization during amplification, improved reaction speed (reduced time-to-positive) and high sensitivity. While standard LAMP, in laboratory settings, can detect even lower amounts but it is prone to non-specific detection and longer reaction times [29,33]. Other studies have also reported that probe-mediated systems perform favorably compared to other isothermal and enhanced PCR methods for tomato begomoviruses, including RPA-LFD [54] and nanoparticle-augmented PCR [15], while eliminating the need for a thermal cycler. In this study, fluorescence signal generation was driven by the intercalating dye, which binds to newly synthesized double stranded DNA during LAMP amplification, resulting in real-time increase in fluorescence proportional to amplicon accumulation. The FITC-labeled hybridization probe binds to the target during amplification. This improves specificity, stabilizes intermediate DNA structures, and facilitates efficient displacement. This enables earlier signal detection and a shorter time-to-positive [55].

The integration of LAMP technology with simple, rapid nucleic acid extraction techniques facilitates swift disease diagnosis in field settings. The time and effort required to prepare DNA samples are often a limiting factor [40]. Numerous methods have been documented for detecting begomoviruses using PCR, including CTAB-based techniques, FTA nucleic acid cards, the toothpick method, and the alkaline lysis approach. Compared with commercial kit-based methods, these methods are favored for their field practicality, and flexibility for varying sample sizes. By contrast, LAMP and RT-LAMP have also been applied with crude nucleic acid extraction, enabling rapid, field-appropriate diagnostics in non-laboratory or low-resource settings [33,50,51,56], potentially enhancing the swift implementation of these approaches in non-laboratory environments, field situations, or low-resource settings. The results of this study corroborate previous findings [33,57], indicating that LAMP is compatible with crude DNA extracts, requires only a water bath for constant-temperature incubation, and can be directly visualized. In this context, we explored an alternative by modifying the APEG lysis technique to extract total nucleic acids from tomato and chili pepper leaves.

In this study, we evaluated a modified alkaline lysis method (APEG + PVP + NaCl), as LAMP requires only minimal DNA template and tolerates crude DNA. This method is suitable for DNA extraction for loop-mediated isothermal amplification because *Bst* polymerase is more tolerant of plant inhibitors than *Taq* polymerase, which is used in PCR. The extracted DNA was adequate for LAMP detection of ToLCV. LAMP assays with the APEG approach have proven effective for tomato mosaic virus [51] and sweet potato viruses [50], and their simplicity aligns with this work [52]. The modified alkaline lysis method yielded good quality DNA (S3 File) and exhibited shorter reaction times than the traditional APEG (Fig 6). To our knowledge, this is the first report to use APEG, PVP, and NaCl in combination for DNA extraction, achieving better threshold times and greater sensitivity (~0.001 fg/μL vs 0.001 ng/μL for PCR). PVP was used to remove phenolic compounds from leaf extracts by co-precipitating polyphenols via hydrogen bonding, effectively clearing them from the lysate. Polyphenols can co-precipitate with DNA and interfere with subsequent processes, including PCR amplification [41]. Sodium chloride facilitates the removal of proteins and polysaccharides associated with DNA [41] and neutralizes the charges on the DNA sugar-phosphate backbone [58]. In addition, a dense macromolecular environment is created in the solution by the neutral, water-soluble polymer polyethylene glycol (PEG), thereby increasing the concentration of DNA molecules [52]. The PEG component of the plant lysis solution also contributes to neutralization after plant cell lysis [59,60]. Moreover, the refined alkaline lysis technique offers numerous advantages over alternative approaches, including the CTAB method. For example, extraction takes less than 5 minutes and is a one-step process conducted in a fully self-contained environment, thereby eliminating the possibility of cross-sample contamination. Furthermore, the extraction process yields a substantial amount of DNA and does not use a fume hood nor generate hazardous waste. The crude extraction step eliminates the need for commercial kits or liquid nitrogen grinding, aligning with field-friendly methods recommended for African cropping systems [11].

The on-site applicability of the assay was evaluated by testing infected tomato and chili pepper leaf samples collected from the field, yielding positive results distinguishable from the negative control reactions involving DNA from a healthy plant sample and nuclease-free water. The diagnosis of ToLCV in asymptomatic samples poses significant challenges, primarily because of low viral titers, which complicate accurate and efficient identification. The analysis of the LAMP and PCR assays demonstrated that LAMP successfully detected ToLCV in asymptomatic tomato and chili pepper samples that were negative by PCR (Figs 9A and 9B, and Table 2), despite the LAMP reactions being conducted on crude DNA extracts isolated using the modified alkaline lysis method. In conjunction with analogous findings from earlier research [33,51–53], this suggests that the LAMP assay demonstrates significant sensitivity and reliability, effectively mitigating disturbance from various substances, such as polysaccharides, lipids, polypeptides, and salts. The recorded false negatives for conventional PCR (Table 2 and S7 File) likely indicate the assay’s lower sensitivity compared to LAMP.

Real-time qPCR was used to confirm the results of the asymptomatic samples that tested positive with LAMP but negative with conventional PCR. The qPCR confirmed all four samples were positive (Figs 10A and 10B), supporting the LAMP findings. LAMP often detects viruses in asymptomatic plants when conventional PCR does not, mainly because LAMP is more sensitive and can handle crude or inhibitor-rich extracts and very low amounts of template [35]. However, conventional PCR is less tolerant of such conditions [22]. LAMP uses four to six primers that bind to six to eight sites, along with a strand-displacing polymerase, which allows it to quickly produce large amounts of product, typically 10⁸ to 10⁹ copies in 30–60 minutes [20], while conventional PCR generates lower copy numbers in the same timeframe. This high amplification efficiency usually means LAMP can detect lower levels of virus than conventional PCR, and its sensitivity is often similar to or better than that of real-time qPCR. As a result, LAMP can detect very low viral loads in asymptomatic plants [51]. Many plant extraction methods co-purify PCR inhibitors, such as phenolics, polysaccharides, and humic acids, which reduce PCR yield. However, LAMP, especially with *Bst* polymerases, is more tolerant of these inhibitors and can produce positive results from quick or crude extractions where conventional PCR might fail [26]. As a result, LAMP is suitable for field screening of asymptomatic samples [33]. Furthermore, real-time and colorimetric LAMP can rapidly detect small increases in product. In contrast, PCR often needs more cycles and cleaner templates to yield a visible band, resulting in slower detection. Accordingly, many studies report that LAMP provides faster results and allows earlier detection [33,51]. In addition, LAMP primers target short, highly conserved regions and frequently include loop primers to accelerate reactions. This design helps capture low-copy targets across variants, improving detection in early or latent infections [51]. For example, in a recent study by Caruso *et al*. [33], real-time LAMP was reported to be approximately 1,000 times more sensitive than conventional PCR, detecting more positives overall, especially asymptomatic plants (LAMP-positive rate 85% vs. PCR 71.6%). Melting-curve analysis corroborated these results. In a similar vein, Kirasi *et al*. [51] found that RT-LAMP detected tomato mosaic virus in 14 field samples, whereas conventional reverse transcriptase PCR detected the virus in only 11. This highlighted RT-LAMP’s greater sensitivity and its ability to reliably detect targets from crude extracts. Caruso *et al*. [61] developed a real-time RT-LAMP test for the nucleocapsid (N) region of tomato spotted wilt virus (TSWV) and assessed its use in both laboratory and field settings. In the field, the test detected TSWV in 37% of samples, compared to 32% with conventional reverse transcriptase-PCR and 29% with real-time RT-PCR, especially in asymptomatic samples.

The probe enhanced LAMP assay demonstrated strong discrimination (AUC = 0.947, 95% CI: 0.898 to 0.997), high sensitivity, and substantial agreement (Cohen’s Kappa = 0.862, 95% Cl: 0.713 to 0.967), suggesting potential suitability for field-based diagnostics. Few plant virus LAMP studies report full statistics, such as ROC curves or likelihood ratios, but available data suggest that LAMP assays usually achieve or exceed 90% sensitivity and specificity. One recent study reported sensitivity of 94.67%, specificity of 100%, and an AUC of 1.0, highlighting excellent performance under optimized conditions [62]. Our results confirmed high sensitivity, consistent with reported trends showing 100- to 1000-fold higher analytical sensitivity for begomoviruses and related plant viruses [33,61]. This was especially evident in detecting low viral levels in asymptomatic samples. LAMP can detect low-level or early infections that PCR might not find, revealing true infections even in asymptomatic samples [24]. The high negative predictive value (1.00) and LR-(0) observed in this study show that LAMP assays are very good at ruling out infections, especially when their sensitivity is close to unity.

LAMP-based methods involve a limited number of manipulations, allowing for effective contamination control even in minimal facilities, particularly in resource-limited field environments. The LAMP method serves as an effective diagnostic tool for the swift and precise detection of Kenyan ToLCV isolates in host plants, particularly in scenarios where the viral titer is minimal in the field. LAMP has improved plant virus diagnostics by enabling rapid, sensitive detection without specialized equipment, making it particularly suitable for field and resource limited settings. The integration of LAMP with the developed modified alkaline lysis DNA extraction method proved to be an effective and innovative tool for diagnosis. Proper control and prevention of disease requires prompt and accurate pathogen detection. Nucleic acid-based testing offers a robust framework for precise and timely disease diagnosis while ensuring the safety of personnel involved. The considerable shortcomings that have historically characterized molecular diagnostics primarily originate from their restricted accessibility, which is attributed to complex protocols, and comparatively low efficiency. The modified alkaline lysis protocol and LAMP can address challenges in nucleic acid-based diagnostics, making them accessible to many, including those in low-resource settings and some developing countries. These unique qualities of the modified protocol, along with its ease of compatibility with LAMP, can enhance its adoption in the diagnosis of many diseases in field settings. The simplicity of the LAMP procedure, along with its accessibility, are the most frequently emphasized innovative characteristics of LAMP [37].

## 5 Conclusion

In this study, we developed a modified APEG lysis approach for fast, effective plant DNA extraction, enabling rapid results and minimizing the need for laboratory equipment in field diagnostics. Unlike traditional methods that are labor-intensive and require sophisticated equipment, the modified alkaline lysis method is simpler, making it ideal for field use and accessible to labs with limited resources. LAMP was used to develop a sensitive and specific test for Kenyan ToLCV isolates detection in a variety of host plants. By combining these two methodologies, we provide a straightforward, efficient diagnostic protocol for both surveillance and phytosanitary certification programs, particularly in resource-constrained settings. Furthermore, this approach can enhance surveillance efficiency when integrated with sensor platforms and can be adapted to diagnose other important plant pathogens. Probe-enhanced LAMP, as demonstrated in this study, enables detection of Kenyan isolates of ToLCV before symptom onset, thereby facilitating earlier intervention. Identifying the pathogen in asymptomatic plants highlights the benefit of pre-symptomatic detection, supporting proactive management. The development of such accessible and dependable diagnostic tools is especially advantageous for laboratories in emerging economies, such as those in Africa, by expanding their capabilities for plant virus identification and epidemiological research and helping to contain future outbreaks.

## Supporting information

S1 File Raw gel images. (PDF)

S2 File GPS readings for sample collection. GPS coordinates for the different sampling regions including Kirinyaga, Embu, Kajiado, Baringo, Narok and Laikipia.
(XLSX)

S3 File Nanodrop readings. An excel file containing 2 sheets: (sheet 1) modified CTAB + column purification; (sheet 2) modified APEG method.
(XLSX)

S4 File Qubit readings. An excel file showing the readings for genomic DNA extracted using modified APEG method and used for qPCR analysis of asymptomatic samples.
(XLSX)

S5 File Ct values and tp values. The values were used to perform statistical analyses and construct the graphs.
(PDF)

S6 File GraphPad analysis files. The file contains multiple Graphpad files for the different statistical analyses which were carried out.
(ZIP)

S7 File Contingency analysis. Table generated on Graphpad showing calculations of diagnostic parameters.
(PDF)

S1 Fig Multiple sequence alignment. An alignment of the sequences used to design the LAMP primers and probes.
(TIFF)

S2 Fig Gel image for genomic DNA. The DNA was extracted using the modified APEG method and used for qPCR analysis of asymptomatic samples.
(TIF)

S3 Fig Real-time qPCR curves. The curves include amplification and melt curves for qPCR validation of the asymptomatic samples.
(TIF)
