# Supplementary material for: Rapid detection of Kenyan tomato leaf curl virus isolates using probe-enhanced loop-mediated isothermal amplification coupled with a modified DNA extraction method
Source: PLoS One. 2026 May 22;21(5):e0349665. doi: 10.1371/journal.pone.0349665 (PMC13196975; doi:10.1371/journal.pone.0349665)
Supplement: S1 File — (PDF) [file pone.0349665.s001.pdf]

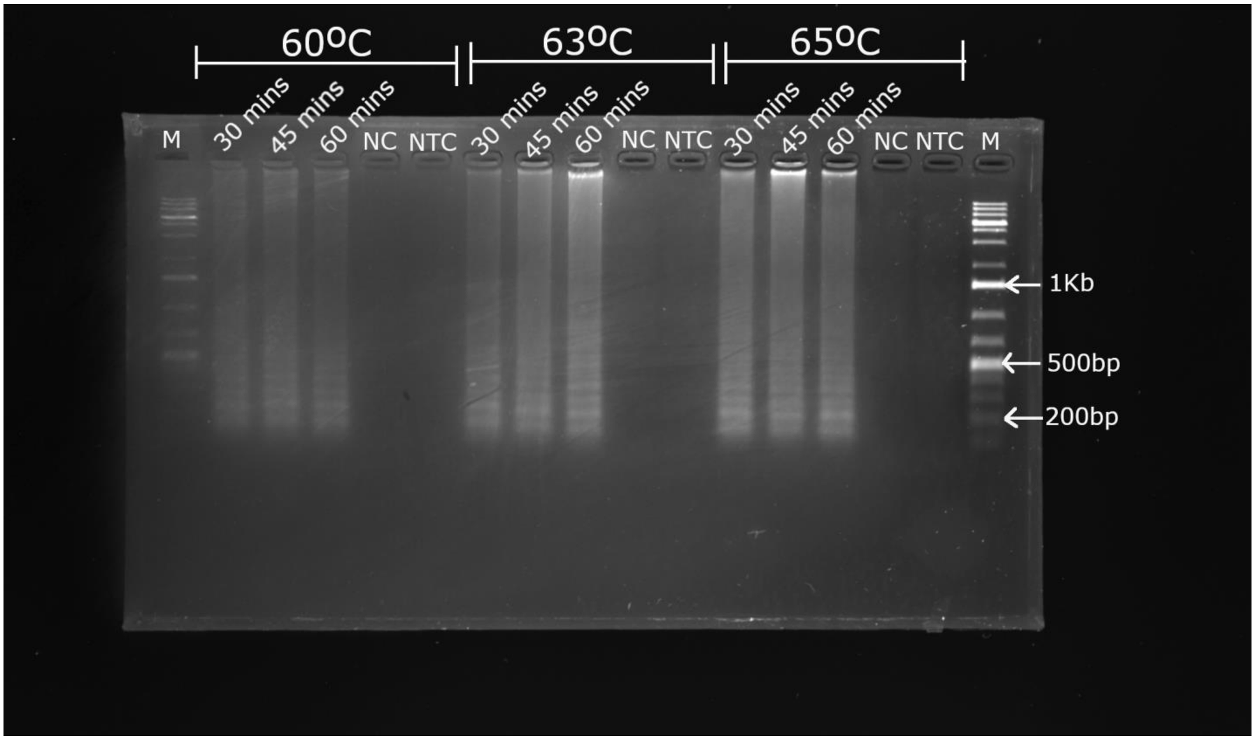

The image was captured using the VILBER E-BOX CX5.TS EDGE (France) UV gel documentation device. Fig 2E was generated from this original image.

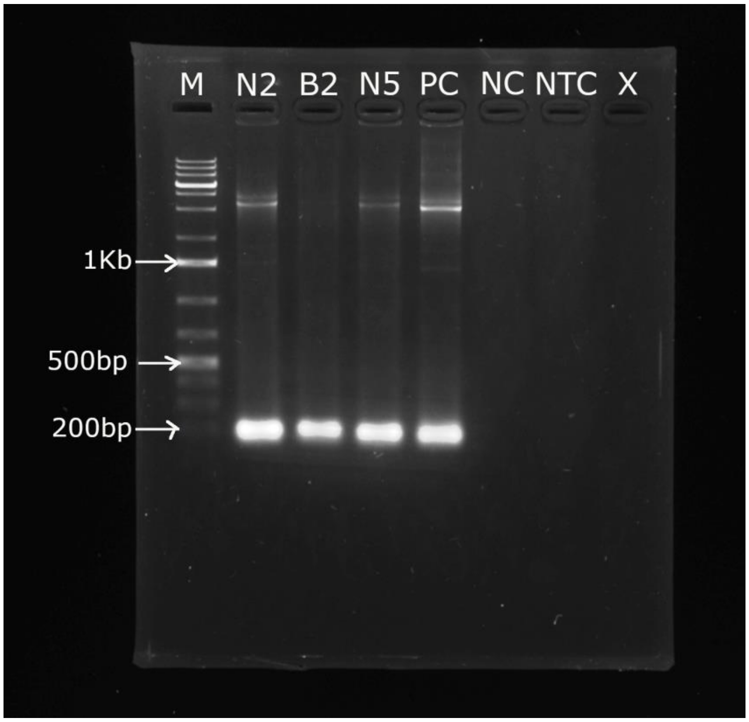

The image was captured using the VILBER E-BOX CX5.TS EDGE (France) UV gel documentation device. Figure 2D was generated from this original image.

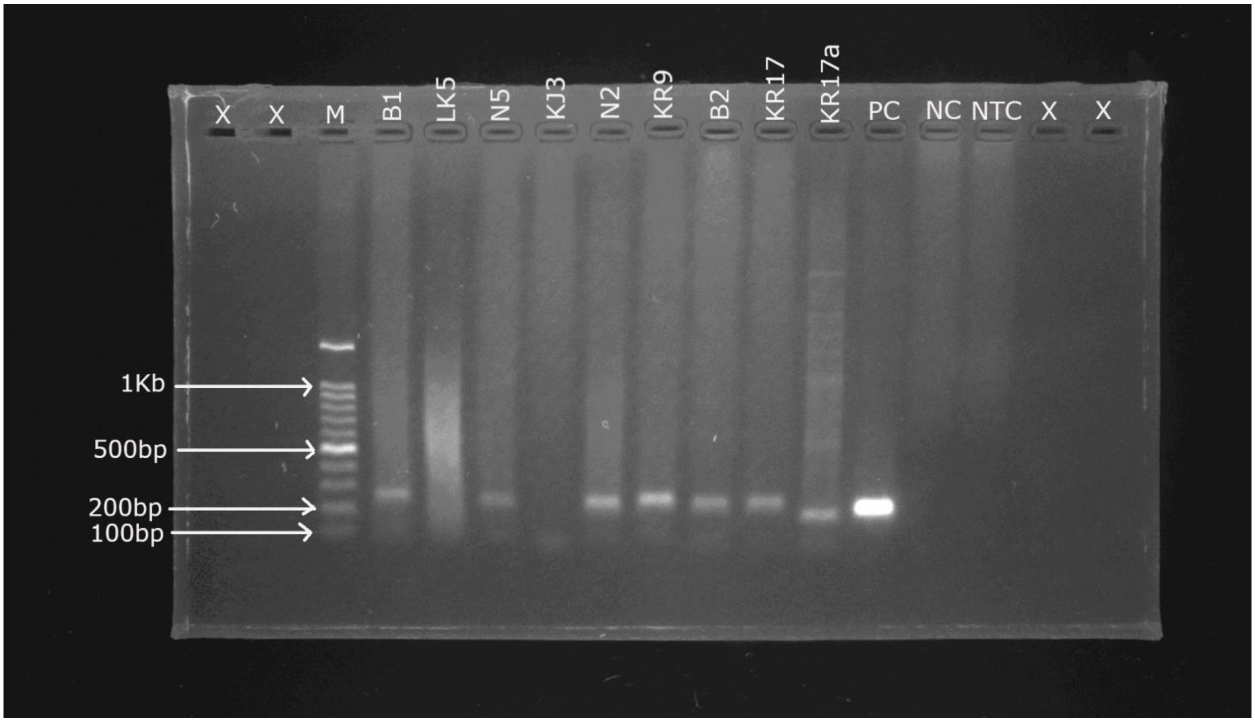

The image was captured using the VILBER E-BOX CX5.TS EDGE (France) UV gel documentation device. Fig 6C was generated from this original image.

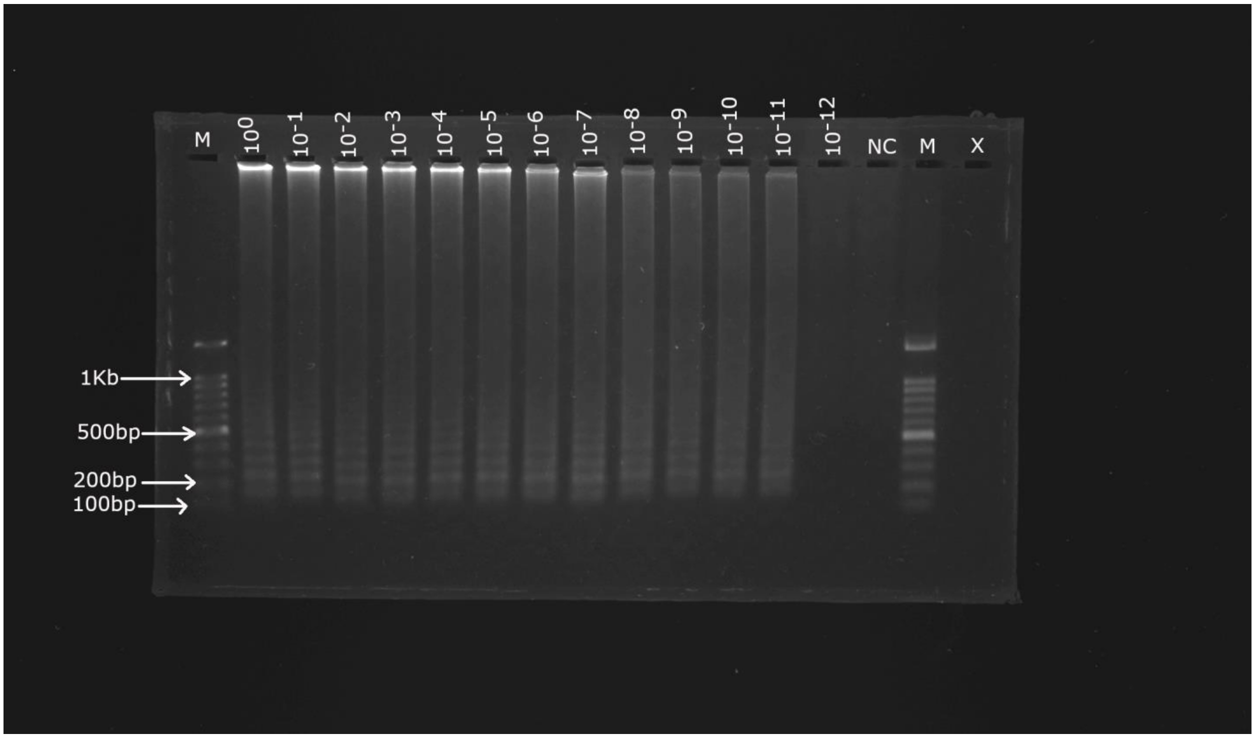

The image was captured using the VILBER E-BOX CX5.TS EDGE (France) UV gel documentation device. Fig 7B was generated from this original image.

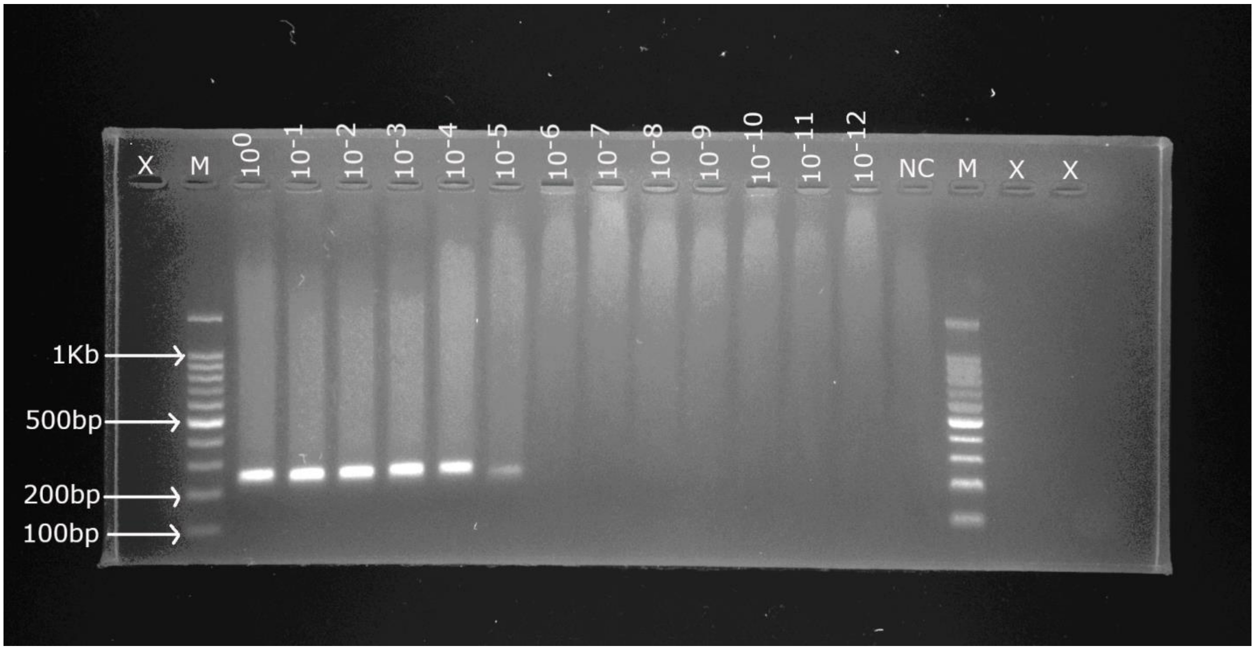

The image was captured using the VILBER E-BOX CX5.TS EDGE (France) UV gel documentation device. Fig 7C was generated from this original image.

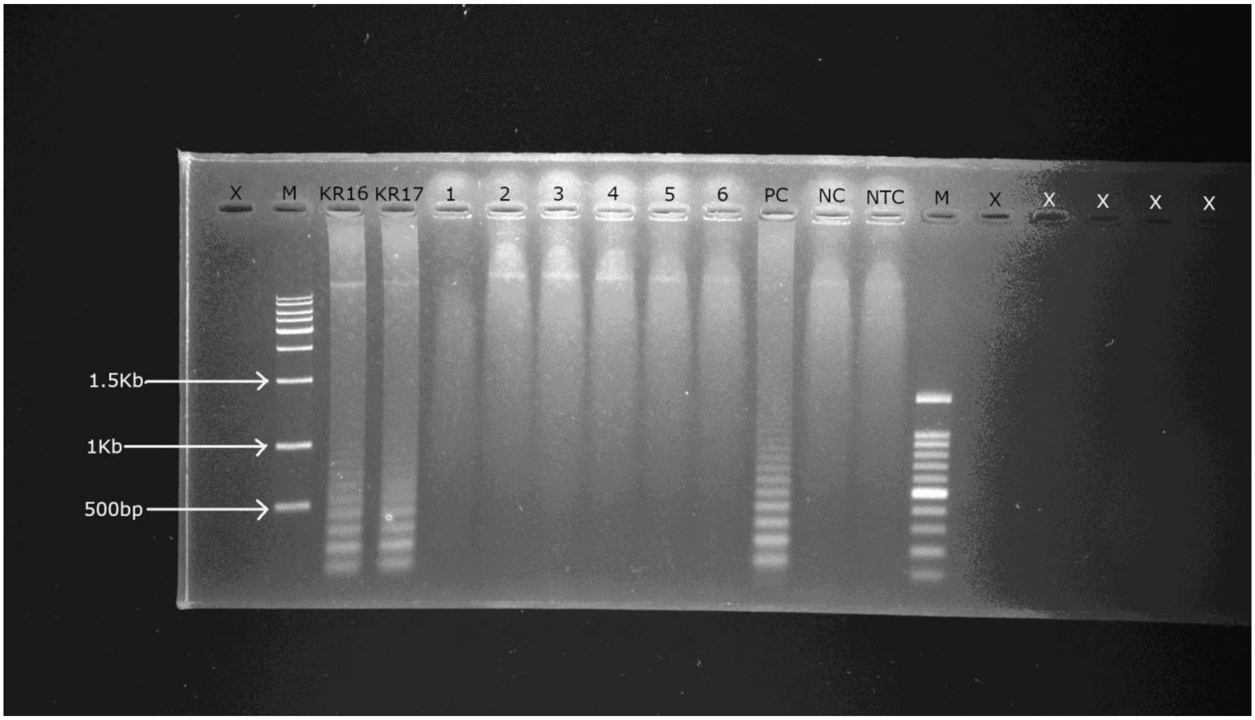

The image was captured using the VILBER E-BOX CX5.TS EDGE (France) UV gel documentation device. Fig 8B was generated from this original image.

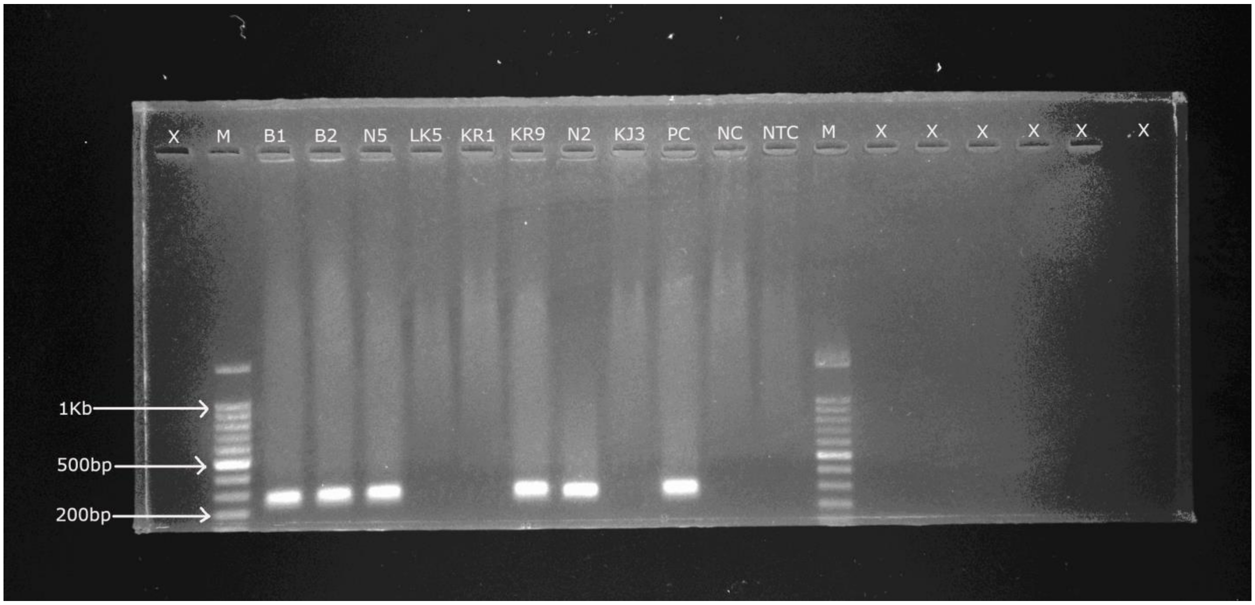

The image was captured using the VILBER E-BOX CX5.TS EDGE (France) UV gel documentation device. Fig 9A was generated from this original image.

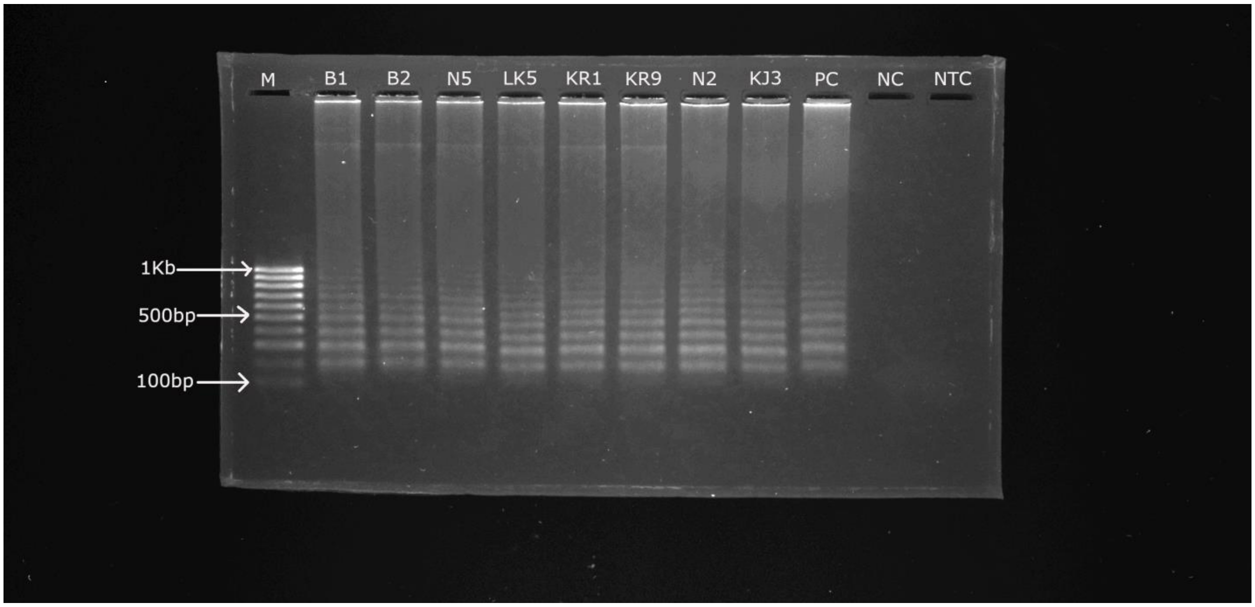

The image was captured using the VILBER E-BOX CX5.TS EDGE (France) UV gel documentation device. Fig 9C was generated from this original image.
