# Supplementary material for: Rapid detection of Kenyan tomato leaf curl virus isolates using probe-enhanced loop-mediated isothermal amplification coupled with a modified DNA extraction method
Source: PLoS One. 2026 May 22;21(5):e0349665. doi: 10.1371/journal.pone.0349665 (PMC13196975; doi:10.1371/journal.pone.0349665)
Supplement: S5 File — The values were used to perform statistical analyses and construct the graphs. (PDF) [file pone.0349665.s005.pdf]

LAMP SCREENING (WITH PROBE)

SAMPLE	CT	MEAN CT	TIME	MEAN TIME
B1	5.78	5.76	2.88	2.8783
B1	5.77		2.885	2.875
B1	5.77		2.87	2.8775
LK5	6.03	5.99	3.015	2.995
LK5	6.02		3.01	2.9875
LK5	5.92		2.96	2.985
N5	5.6	5.67	2.8	2.835
N5	5.6		2.8	2.8525
N5	5.81		2.905	2.8525
KR9	5.64	5.69	2.82	2.843333
KR9	5,63		2.815	2.8175
KR9	5.79		2.895	2.855
N2	5.88	5.9	2.94	2.948333
N2	6.06		3.03	2.9075
N2	5.75		2.875	2.9525
KR1	6	5.95	3	2.973333
KR1	5.91		2.955	2.9825
KR1	5.93		2.965	2.96
B2	6.03	5.98	3.015	2.99
B2	5.88		2.94	3.015
B2	6.03		3.015	2.9775
KJ3	5.98	5.9	2.99	2.95
KJ3	5.85		2.925	2.9625
KJ3	5.87		2.935	2.93
PC	5.79	5.79	2.895	2.8925
PC	5.57		2.785	2.9475
PC	6		3	2.8925
NC	30.67	30.67		
NC	NO CT			
NC	NO CT			
NTC	NO CT			
NTC	NO CT			
NTC	NO CT			

WITH/WITHOUT LOOP PRIMERS
N- WITH LOOP PRIMERS
L-WITHOUT LOOP PRIMERS

SAMPLE	CT	MEAN CT		TIME	MEAN TIME
N1	11.61	9.716667		5.805	4.8583
N1	8.22			4.11	5.2325
N1	9.32			4.66	4.385
N2	11.3	12.00667		5.65	6.0033
N2	12.26			6.13	5.94
N2	12.46			6.23	6.18
N3	11.11	11.06		5.555	5.53
N3	11.03			5.515	5.5375
N3	11.04			5.52	5.5175
N4	10.88	11.35333		5.44	5.6767
N4	11.7			5.85	5.59
N4	11.48			5.74	5.795
N5	15.22	12.54		7.61	6.27
N5	15.08			7.54	5.635
N5	7.32			3.66	5.6
PC	12.68	11.45667		6.34	5.7283
PC	9.46			4.73	6.2275
PC	12.23			6.115	5.4225
L1	32.02	32.64333		16.01	16.3217
L1	32.39			16.195	16.385
L1	33.52			16.76	16.4775
L2	37	40.33333		18.5	20.1667
L2	42.12			21.06	19.72
L2	41.88			20.94	21
L3	25.5	34.07		12.75	17.035
L3	36.69			18.345	16.38
L3	40.02			20.01	19.1775
L4	50.86	30.35		25.43	15.175
L4	19.18			9.59	17.9675
L4	21.01			10.505	10.0475
L5	33.71	35.27333		16.855	17.6367

L5	36.29			18.145	17.3825
L5	35.82			17.91	18.0275
PC	34.59	37.02		17.295	18.51
PC	39.81			19.905	17.8125
PC	36.66			18.33	19.1175
NC	NO CT				
NC	NO CT				
NC	NO CT				
NTC	NO CT				
NTC	NO CT				
NTC	NO CT				

APEG OPTIMSATION (NaCL CONCENTRATION)

SAMPLE	NaCl CONC
SI	0.05M
S2	0.5M
S3	0.1M
S4	1.0M
S5	0.8M

APEG +PVP + NaCL

SAMPLE	Ct	MEAN Ct	TIME	MEAN TIME
S1	15.49		7.745	7.485
S1	15.06		7.53	7.638
S1	14.36		7.18	7.4625
S2	14.79		7.395	7.165
S2	14.27		7.135	7.05
S2	13.93		6.965	7.18
S3	15.08		7.54	7.542
S3	15.03		7.515	7.528
S3	15.14		7.57	7.555
S4	11.11		5.55	5.48
S4	11.39		5.695	5.445
S4	10.39		5.195	5.3725
S5	11.05		5.525	5.255
S5	10.02		5.01	5.12
S5	10.46		5.23	5.3775

KR17	15.01		7.505	7.49
KR17	14.93		7.465	7.485
KR17	15		7.5	7.5025
PC	16.32		8.16	8.237
PC	16.6		8.3	8.23
PC	16.5		8.25	8.205
NC	NO CT			
NC	NO CT			
NC	NO CT			
NTC	NO CT			
NTC	NO CT			
NTC	NO CT			

LAMP SCREENING (WITHOUT PROBE)

SAMPLE	CT	MEAN CT	TIME	MEAN TIME
B1	14.59	14.62	7.295	7.311667
B1	14.8		7.4	7.2675
B1	14.48		7.24	7.32
LK5	15.03	15.07	7.515	7.535
LK5	15.01		7.505	7.55
LK5	15.17		7.585	7.545
N5	16.15	15.88	8.075	7.94
N5	15.49		7.745	7.91
N5	16		8	7.8725
KR9	11.06	10.85	5.53	5.428333
KR9	10.93		5.465	5.41
KR9	10.58		5.29	5.3775
N2	13.02	12.99	6.51	6.493333
N2	12.93		6.465	6.5075
N2	13.01		6.505	6.485
KR1	16.73	16.56	8.365	8.278333
KR1	16.39		8.195	8.32
KR1	16.55		8.275	8.235
B2	18.26	18.12	9.13	9.063333

B2	18.21		9.105	9.0425
B2	17.91		8.955	9.03
KJ3	17.09	17.21	8.545	8.601667
KJ3	17.32		8.66	8.5725
KJ3	17.2		8.6	8.63
PC	11.85	12.76	5.925	6.381667
PC	13.14		6.57	6.2875
PC	13.3		6.65	6.61
NC	NO CT			
NC	NO CT			
NC	NO CT			
NTC	NO CT			
NTC	NO CT			
NTC	NO CT			

Different Extraction Methods

X1 - APEG+PVP

X2 - APEG+PVP+NaCL

X3 - APEG

X4 - APEG+NaCL

SAMPLE	CT	MEAN CT	TIME	MEAN TIME
X1	13.68	13.84	6.84	6.92
X1	13.97		6.985	6.89
X1	13.87		6.935	6.96
X2	11.33	9.55	5.665	4.77
X2	10.68		5.34	4.49
X2	6.63		3.315	4.33
X3	15.62	15.38	7.81	7.69
X3	15.09		7.545	7.77
X3	15.44		7.72	7.63
X4	11.23	11.48	5.615	5.74
X4	11.56		5.78	5.8025
X4	11.65		5.825	5.70
KR17	15.66	15.13	7.83	7.57
KR17	13.49		6.745	7.98
KR17	16.24		8.12	7.43
PC	16.56	16.22	8.28	8.11

PC	16.6		8.3	8.0125
PC	15.49		7.745	8.0225
NC	NO CT			
NC	NO CT			
NC	NO CT			
NTC	NO CT			
NTC	NO CT			
NTC	NO CT			

Real time qPCR

S1 - KR1
S2 - LK5
S3 - KJ3
S4 - KJ6
S5 - B1
S6 - N5
PC

Sample ID	Ct	Mean Ct	Mean	SD
KR1	21.71	21.81	21.81333	0.040893
KR1	21.82	21.765		
KR1	21.91	21.865		
LK5	21.29	21.77	21.77	0.195959
LK5	21.77	21.53		
LK5	22.25	22.01		
KJ3	21.41	21.51	21.51	0.036742
KJ3	21.52	21.465		
KJ3	21.59	21.555		
KJ6	21.74	22.3	22.21667	0.331872
KJ6	21.81	21.775		
KJ6	23.34	22.575		
B1	21.18	21.41	21.44667	0.085667
B1	21.55	21.365		
B1	21.58	21.565		
N5	8.12	8.22	8.213333	0.045154
N5	8.19	8.155		
N5	8.34	8.265		

PC		8.79	8.86	8.863333	0.024608
PC		8.88	8.835		
PC		8.91	8.895		
NC	NO CT				
NC	NO CT				
NC	NO CT				
NTC	NO CT				
NTC	NO CT				
NTC	NO CT				
