# Supplementary material for: Rapid detection of Kenyan tomato leaf curl virus isolates using probe-enhanced loop-mediated isothermal amplification coupled with a modified DNA extraction method
Source: PLoS One. 2026 May 22;21(5):e0349665. doi: 10.1371/journal.pone.0349665 (PMC13196975; doi:10.1371/journal.pone.0349665)
Supplement: S7 File — Table generated on Graphpad showing calculations of diagnostic parameters. (PDF) [file pone.0349665.s007.pdf]

Contingency		A	B	C
1	**Table Analyzed**	Data 1		
2				
3	**P value and statistical significance**			
4	Test	Fisher's exact test		
5	P value	<0.0001		
6	P value summary	****		
7	One- or two-sided	Two-sided		
8	Statistically significant (P < 0.05)?	Yes		
9				
10	**Effect size**	**Value**	**95% CI**	
11	Sensitivity	1.000	0.8513 to 1.000	
12	Specificity	0.8947	0.7587 to 0.9583	
13	Positive Predictive Value	0.8462	0.6647 to 0.9385	
14	Negative Predictive Value	1.000	0.8985 to 1.000	
15	Likelihood Ratio	9.500		
16				
17	**Methods used to compute CIs**			
18	Sensitivity, specificity, etc.	Wilson-Brown		
19				
20	**Data analyzed**	**PCR +**	**PCR -**	**Total**
21	LAMP +	22	4	26
22	LAMP -	0	34	34
23	Total	22	38	60
24				
25	**Percentage of row total**	**PCR +**	**PCR -**	
26	LAMP +	84.62%	15.38%	
27	LAMP -	0.00%	100.00%	
28				
29	**Percentage of column total**	**PCR +**	**PCR -**	
30	LAMP +	100.00%	10.53%	
31	LAMP -	0.00%	89.47%	
32				
33	**Percentage of grand total**	**PCR +**	**PCR -**	
34	LAMP +	36.67%	6.67%	
35	LAMP -	0.00%	56.67%	
