# Supplementary figures and images for: Rapid detection of Kenyan tomato leaf curl virus isolates using probe-enhanced loop-mediated isothermal amplification coupled with a modified DNA extraction method
Source: PLoS One. 2026 May 22;21(5):e0349665. doi: 10.1371/journal.pone.0349665 (PMC13196975; doi:10.1371/journal.pone.0349665)

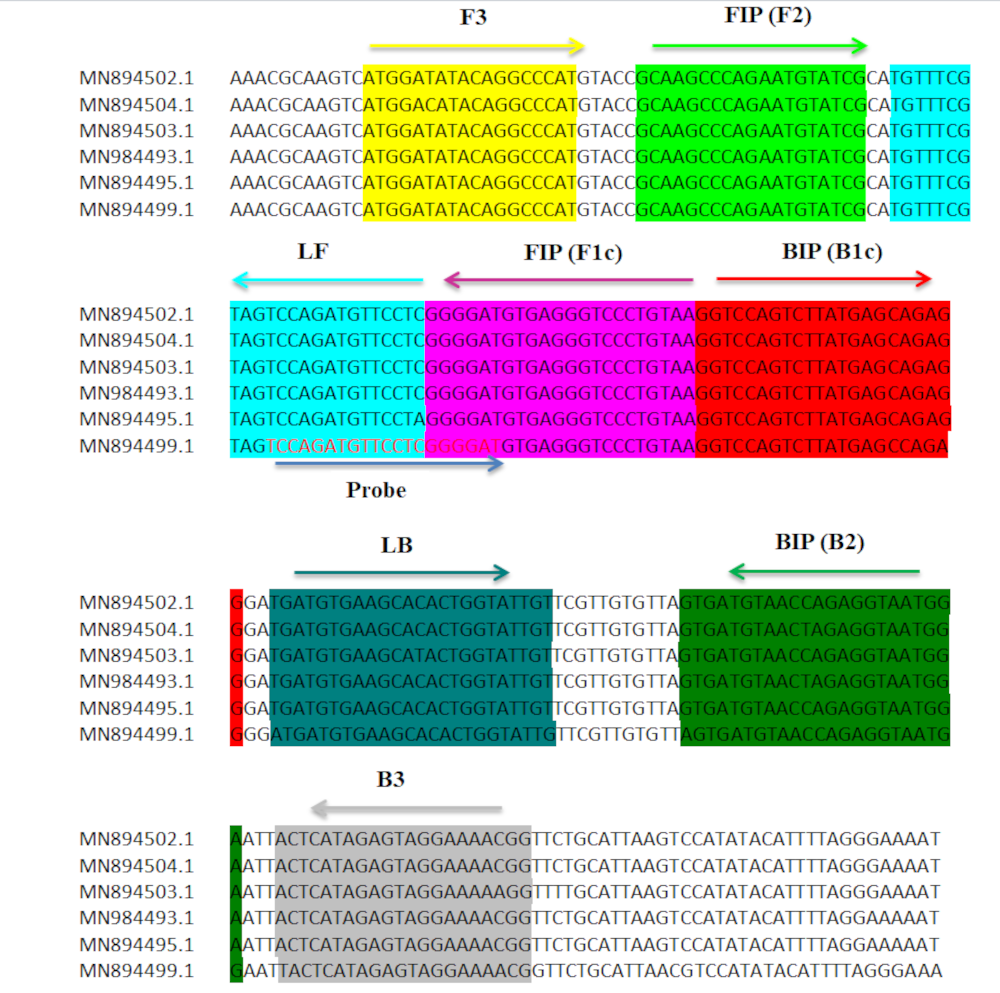

Supplement: S1 Fig — An alignment of the sequences used to design the LAMP primers and probes. (TIFF) [file pone.0349665.s008.tiff]

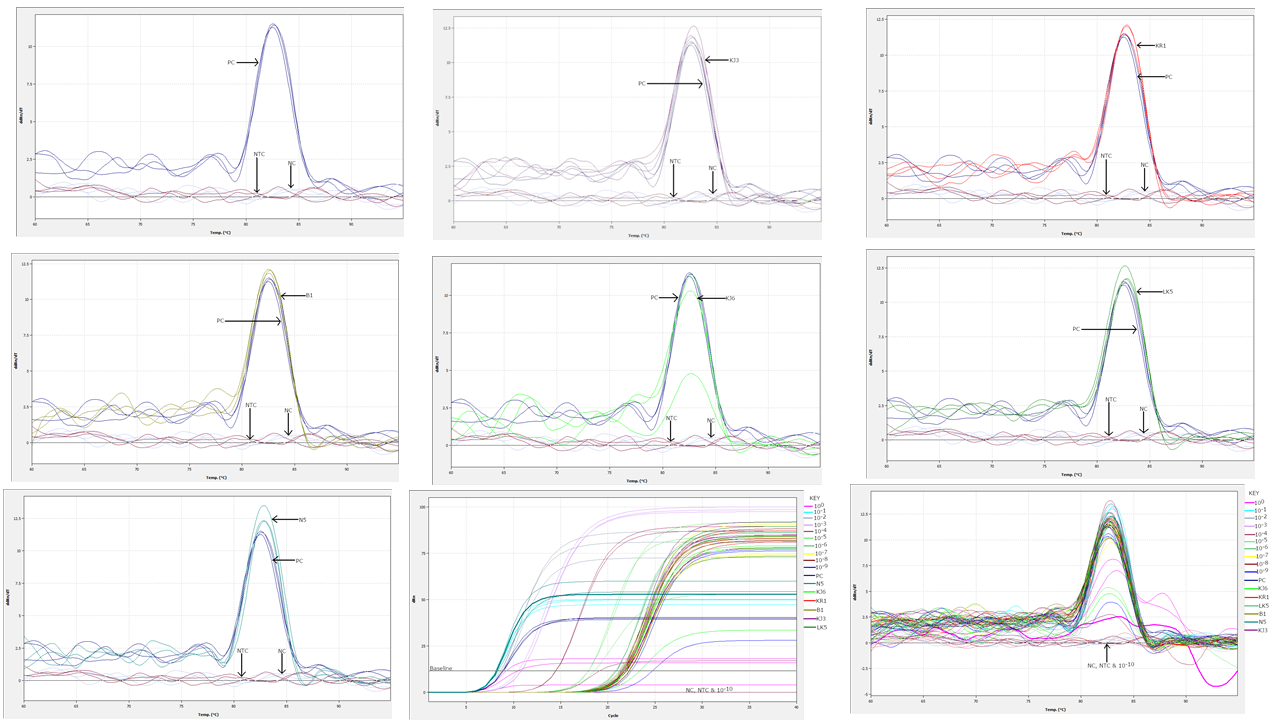

Supplement: S3 Fig — The curves include amplification and melt curves for qPCR validation of the asymptomatic samples. (TIF) [file pone.0349665.s010.tif]
